# Australian guideline on diagnosis and management of peripheral artery disease: part of the 2021 Australian evidence-based guidelines for diabetes-related foot disease

**DOI:** 10.1186/s13047-022-00550-7

**Published:** 2022-07-05

**Authors:** Vivienne Chuter, Frank Quigley, Patrik Tosenovsky, Jens Carsten Ritter, James Charles, Jane Cheney, Robert Fitridge, on behalf of the Australian Diabetes-related Foot Disease Guidelines & Pathways Project, Stephen Twigg, Peter Lazzarini, Anita Raspovic, Jenny Prentice, Robert Commons, Robert Fitridge, James Charles, Jane Cheney

**Affiliations:** 1 School of Health Sciences, Western Sydney University, Campbelltown, Australia; 2 Discipline of Podiatry, University of Newcastle, Ourimbah, Australia; 3 The Mater Hospital, Townsville, Australia; 4 Department of Vascular & Endovascular Surgery, Royal Perth Hospital, Wellington Street, Perth, Australia; 5 Department of Vascular Surgery, Fiona Stanley Hospital, Perth, Australia; 6 Curtin University, School of Medicine, Perth, Australia; 7 First Peoples Health Unit, Griffith University, Gold Coast, Australia; 8 Diabetes Victoria, Melbourne, Australia; 9 Discipline of Surgery, The University of Adelaide, Adelaide, Australia; 10 Vascular and Endovascular Service, Central Adelaide Local Health Network, Adelaide, Australia; 11 Diabetes Feet Australia, Brisbane, Australia; 12 Australian Diabetes Society, Sydney, Australia

**Keywords:** Diabetes feet, Peripheral artery disease, Foot ulcer, Guidelines, Diagnosis, Revascularisation

## Abstract

**Background:**

Peripheral artery disease (PAD) is implicated in up to 50% of diabetes-related foot ulcers (DFU) and significantly contributes to morbidity and mortality in this population. An evidence-based guideline that is relevant to the national context including consideration of the unique geographical and health care system differences between Australia and other countries, and delivery of culturally safe care to First Nations people, is urgently required to improve outcomes for patients with PAD and DFU in Australia. We aimed to identify and adapt current international guidelines for diagnosis and management of patients with PAD and DFU to develop an updated Australian guideline.

**Methods:**

Using a panel of national content experts and the National Health and Medical Research Council procedures, the 2019 International Working Group on the Diabetic Foot (IWGDF) guidelines were adapted to the Australian context. The guideline adaptation frameworks ADAPTE and Grading of Recommendations Assessment, Development and Evaluation (GRADE) were applied to the IWGDF guideline for PAD by the expert panel. Recommendations were then adopted, adapted or excluded, and specific considerations for implementation, population subgroups, monitoring and future research in Australia were developed with accompanying clinical pathways provided to support guideline implementation.

**Results:**

Of the 17 recommendations from the IWGDF Guideline on diagnosis, prognosis and management of PAD in patients with diabetes with and without foot ulcers, 16 were adopted for the Australian guideline and one recommendation was adapted due to the original recommendation lacking feasibility in the Australian context. In Australia we recommend all people with diabetes and DFU undergo clinical assessment for PAD with accompanying bedside testing. Further vascular imaging and possible need for revascularisation should be considered for all patients with non-healing DFU irrespective of bedside results. All centres treating DFU should have expertise in, and/or rapid access to facilities necessary to diagnose and treat PAD, and should provide multidisciplinary care post-operatively, including implementation of intensive cardiovascular risk management.

**Conclusions:**

A guideline containing 17 recommendations for the diagnosis and management of PAD for Australian patients with DFU was developed with accompanying clinical pathways. As part of the adaptation of the IWGDF guideline to the Australian context, recommendations are supported by considerations for implementation, monitoring, and future research priorities, and in relation to specific subgroups including Aboriginal and Torres Strait Islander people, and geographically remote people. This manuscript has been published online in full with the authorisation of Diabetes Feet Australia and can be found on the Diabetes Feet Australia website: https://www.diabetesfeetaustralia.org/new-guidelines/.

**Supplementary Information:**

The online version contains supplementary material available at 10.1186/s13047-022-00550-7.

## Background

Current global estimates are that diabetes affects 1 in 11 adults (463 million people) [1]. This is expected to increase to 1 in 10, or 700 million people, by 2045 [1]. Diabetes is associated with significant risk of diabetes-related foot disease (DFD) including a life-time incidence of foot ulcer of up to 34% and it is the leading cause of amputation [2]. Up to 50,000 Australians are estimated to be affected by diabetes-related foot ulcer (DFU) with a further 300,000 living at risk of DFU development. DFD occurs more frequently in Aboriginal and Torres Strait Islander people and outcomes are more severe [3], with amputation rates up to 38 times higher than in age-matched non-Indigenous counterparts [4]. Reducing rates of DFD for all Australians is essential to prevent avoidable amputations and reduce the associated AUD$1.6 billion in annual health care costs [5, 6]. As reflected by key outcomes identified in the 2020 ‘Closing the Gap in Partnership’ agreement, there is an urgent need to prioritise and achieve better health outcomes for Aboriginal and Torres Strait Islander people to protect against the devastating consequences of DFD in this population [3, 7].

Peripheral artery disease (PAD) is estimated to affect up to 15% of Australian adults [8]. Diabetes is associated with a four-fold increase in incidence of PAD, independent of other risk factors [9]. PAD is estimated to be present in up to 50% of DFU and to be an independent risk factor in their development [10, 11]. PAD commonly co-exists with systemic atherosclerosis and underlying generalised endothelial dysfunction due to vascular inflammation and an abnormal metabolic state [9, 12]. Together these changes increase the risk of cardiovascular morbidity and mortality significantly [13]. When associated with diabetes, PAD is also more diffuse with increased involvement of tibial arteries [14], greater severity of the disease process, higher likelihood of distal ischaemic ulcer and extensive tissue loss, and increased risk of amputation [15]. Early diagnosis and treatment of PAD in people with DFU is critical due to the increased risk of non-healing, infection and amputation, as well as elevated rate of cardiovascular complications such as myocardial infarction and stroke, and a five-year mortality rate of more than 50% [16–20].

Despite the severity of the outcomes of PAD in people with diabetes, and particularly in those with DFU, there are limited data to determine best practice treatments for this specific population [21, 22]. The majority of current evidence for diagnosis and management of PAD is garnered from the general population and does not account for the multi-system nature of diabetes, and the impact of related complications on healing and amputation outcomes [21]. Multiple diagnostic, surgical, and conservative management options are available to treat PAD and chronic limb-threatening ischaemia [21]. However, to determine best practice in a diabetes population, evidence-based guidelines that provide recommendations specifically for the diagnosis and management of PAD in patients with diabetes and DFU have been developed internationally [21].

In Australia, national evidence-based guidelines for the assessment and management of DFD have not been published since 2011 [23]. Several international evidence-based DFD guidelines have been published recently [17, 24, 25]. However, parts of these guidelines may not be appropriate or applicable in an Australian clinical setting. This is due to the unique geographical and health care system differences between Australia and other parts of the world. Further, the diverse population groups within Australia such as Aboriginal and Torres Strait Islander people, and those in geographically remote areas, require specific focus [26]. To develop new, high quality, evidenced-based guidelines for an Australian context is estimated to cost $AU1 million and significant development time preventing rapid translation to practice [26]. Therefore, to develop a suitable national guideline for the assessment and management of PAD in people with DFU, we aimed to systematically identify and adapt suitable international guidelines.

## Methods

The methodology for this guideline is detailed in an accompanying paper authored by the Australian DFD Guidelines working group [27]. We followed the eight overarching National Health and Medical Research Council (NHMRC) recommendations for adapting source guidelines as described previously [28–30]. The initial three steps of these recommendations include defining the scope, identifying potential source guidelines, and assessing their suitability. The outcomes from this process are described in the development paper [27]. Through this process the 2019 International Working Group on the Diabetic Foot (IWGDF) guidelines were identified as the only suitable source guideline [27]. This guideline and the subsequent five NHMRC steps for adapting source guidelines are the subject of this manuscript and are outlined below.

A national expert panel (‘the authors’) was established by the Australian DFD Guidelines Working Group to develop this PAD guideline. The panel consists of recognised multi-disciplinary (inter) national experts in PAD for people with DFU along with consumer, end-user, and Aboriginal and Torres Strait Islander DFD experts [27]. The authors were provided all PAD recommendations (and all supporting rationale and evidence) from the IWGDF guidelines [19, 21, 31] to consider as the basis for developing this guideline [27].

Using a customised 7-item ADAPTE evaluation form, pairs of panel members independently screened each IWGDF PAD recommendation (and rationale) for their quality of evidence, strength of recommendation, and acceptability and applicability in the Australian national context [27, 30]. All panel members participated in this process. Disagreements between the two panel members on any ratings were discussed until consensus was reached. If required, a third panel member arbitrated disagreements. The panel then met to discuss and gain consensus decisions on the ratings for all recommendations. Any recommendations in which the panel were ‘certain’ that all items agreed with the quality of evidence and strength of recommendation made by IWGDF, and they were acceptable and applicable in the Australian national context were adopted. Recommendations that the panel rated as being ‘unsure’ or ‘not certain’ of the quality of evidence, strength of recommendation, or being acceptable or applicable in the Australia context, were referred to be further assessed in the next stage [27, 30].

The GRADE Evidence to Decision (EtD) tool for clinical recommendations was used to assess recommendations requiring full assessment [27, 29, 32, 33]. This process required one panel member to extract and populate the EtD tool with supporting text for the recommendation from the IWGDF PAD guideline and systematic reviews [19, 21, 31] for eight EtD criteria: the problem, desirable effects, undesirable effects, quality (or certainty) of evidence, values (of importance of outcomes), balance of effects, acceptability and applicability [27, 29, 32, 33]. The populated EtD tool was cross-checked by a second panel member and any disagreements were discussed until a consensus was reached. Following this, an additional panel member assessed the completed EtD tool which was then checked by another panel member with any disagreements discussed until consensus achieved. The panel then met to discuss and gain consensus on their summary judgements for the eight EtD criteria [32, 33] followed by a direct comparison with the IWGDF judgements [27, 29].

Based on the level of agreement between the panel and IWGDF summary judgements, the panel then decided to adopt, adapt, or exclude the recommendation for the Australian national context [27, 29]. A recommendation was ‘adopted’ if there were no substantial differences between the panel and IWGDF summary judgements. Recommendations were ‘adapted’ if there were substantial differences between the panel and IWGDF summary judgements, or ‘excluded’ if there were substantial differences and/or the panel concluded the recommendation was not acceptable or not applicable in Australia [27, 29]. Disagreements within the panel were discussed until consensus was reached or, if that was not possible, by discussing with the Guideline Working Group until consensus was reached.

Those recommendations the panel decided to ‘adapt’ had their quality of evidence, strength of recommendation rating [29, 32, 33], and written recommendations re-evaluated, via consensus based on the panel’s summary judgements [27, 29]. The panel rated the quality of evidence as per the GRADE system [34, 35]. A ‘high’ quality rating was determined if the panel was very confident that the findings from the supporting evidence were from studies with low risk of bias that reported consistent effects and further research was unlikely to change that confidence. A ‘moderate’ quality rating was determined if the panel had moderate confidence in the risk of bias or consistency of effects and additional research was likely to impact that confidence further. A ‘low’ quality rating was determined if the panel had limited confidence in the risk of bias and inconsistency of effects and further research was very likely to impact confidence. Finally, a ‘very low’ quality rating was determined if the panel had very little confidence in the available supporting evidence [32, 33]. The panel also rated the strength of recommendation based on GRADE system. This required the panel members to weigh up the balance of effects, quality of evidence, values, and applicability and acceptability [32, 33] in the Australian national context [27]. The panel provided a ‘strong’ recommendation if there was clearly a moderate-to-large difference in the balance of effects between the intervention compared with the control. The panel provided a ‘weak’ recommendation if there was an uncertainty and/or mild-to-moderate difference between the intervention and control [32, 33]. The panel then re-wrote any ‘adapted’ recommendation to be clear, specific and unambiguous for the Australian context, as per GRADE requirements [35].

For each recommendation the panel drafted the decision rationale, summary justifications for their judgements, detailed justifications for important EtD criteria (if the recommendation was fully assessed), and considerations for implementation (including for geographically remote and Aboriginal and Torres Strait Islander people), monitoring and potential future research priorities [29, 32, 33] in the Australian national context [27]. For recommendations relating to diagnostic testing consideration was given to diagnostic accuracy, direct benefits and adverse effects or burden of the test, as well implications for management. A consultation draft manuscript, including all recommendations (and rationale) for the PAD guideline, was developed by the panel and distributed for public consultation [27]. The consultation draft manuscript of the PAD guideline underwent a formal one-month public consultation period using a customised consultation survey from ADAPTE [27, 30]. All relevant survey and written feedback from the consultation period was collated and analysed, and the manuscript was revised accordingly by the panel members [27, 30].

The panel then used the finalised recommendations to develop two PAD clinical pathways, one for patients with DFU and one for those with diabetes only [27]. The pathway development methodology followed the 10-step process for developing and implementing clinical pathways [18] and has been outlined in detail in the accompanying development of the guideline paper [27]. The purpose of the clinical pathways is to assist the implementation of the PAD recommendations by the multiple health professional disciplines caring for Australians with DFU in secondary and tertiary health care settings [27].

Finally, the panel members sought endorsement from the Guidelines Development Working Group and relevant peak national bodies, including the Australian and New Zealand Society for Vascular Surgery, Diabetes Australia, and the Australian Podiatry Association before the final guideline was released [27].

## Results

A systematic evaluation of the 17 IWGDF recommendations for the diagnosis, prognosis and management of PAD in patients with diabetes-related foot ulcers was conducted to determine their quality of evidence, strength of recommendation, acceptability and applicability to the Australian context. After screening, one of the 17 recommendations required additional full assessment (Table 1). Following full assessment, the recommendation was adapted to be considered acceptable and applicable in the Australian health context. The reasons for adaptation related to differing availability of expertise and equipment in more geographically isolated areas. The other 16 recommendations were considered applicable and acceptable and were adopted (Table 2). The adopted and adapted IWGDF guidelines are summarised in Table 3.

**Table 1:** Summary of screening ratings for acceptability and applicability in the Australian context for all IWGDF PAD recommendations

Recommendation	Acceptability			Applicability				Full assessment	Comments
	1	2	3	4	5	6	7		
1	+	+	+	+	+	+	+	No	
2	+	+	+	+	+	+	+	No	
3	+	+	+	+	+	+	+	No	
4	+	+	+	+	+	+	+	No	
5	+	+	+	+	+	+	+	No	
6	+	+	+	+	+	+	+	No	
7	+	+	+	+	+	+	+	No	
8	+	+	+	+	+	+	+	No	
9	+	+	+	+	+	+	+	No	
11	+	+	+	+	+	+	+	No	
12	+	+	+	+	+	+	+	No	
13	+	+	+	+	?	?	+	Yes	Assess equipment availability & availability of expertise
14	+	+	+	+	+	+	+	No	
15	+	+	+	+	+	+	+	No	
16	+	+	+	+	+	+	+	No	
17	+	+	+	+	+	+	+	No	
Total	**17**	**17**	**17**	**17**	**16**	**16**	**17**	**1**	
%	**100%**	**100%**	**100%**	**100%**	**94%**	**100%**	**100%**	**6%**	

+, yes item is met; −, no item is not met;? unsure if item is met

**Table 2:** Summary of final panel judgements compared with IWGDF judgements for all IWGDF PAD recommendations

No	Problem	Desirable effects	Undesirable effects	Quality of evidence	Values	Balance of effects	Acceptability	Applicability/feasibility	Decision	Comment
1	+	+	+	+	+	+	+	+	Adopted	Adopted in ADAPTE screening
2	+	+	+	+	+	+	+	+	Adopted	Adopted in ADAPTE screening
3	+	+	+	+	+	+	+	+	Adopted	Adopted in ADAPTE screening
4	+	+	+	+	+	+	+	+	Adopted	Adopted in ADAPTE screening
5	+	+	+	+	+	+	+	+	Adopted	Adopted in ADAPTE screening
6	+	+	+	+	+	+	+	+	Adopted	Adopted in ADAPTE screening
7	+	+	+	+	+	+	+	+	Adopted	Adopted in ADAPTE screening
8	+	+	+	+	+	+	+	+	Adopted	Adopted in ADAPTE screening
9	+	+	+	+	+	+	+	+	Adopted	Adopted in ADAPTE screening
10	+	+	+	+	+	+	+	+	Adopted	Adopted in ADAPTE screening
11	+	+	+	+	+	+	+	+	Adopted	Adopted in ADAPTE screening
12	+	+	+	+	+	+	+	+	Adopted	Adopted in ADAPTE screening
13	+	+	+	+	+	+	+	?	Adapted	Adapted: assess equipment availability & availability of expertise
14	+	+	+	+	+	+	+	+	Adopted	Adopted in ADAPTE screening
15	+	+	+	+	+	+	+	+	Adopted	Adopted in ADAPTE screening
16	+	+	+	+	+	+	+	+	Adopted	Adopted in ADAPTE screening
17	+	+	+	+	+	+	+	+	Adopted	Adopted in ADAPTE screening

+, panel agreed with original IWGDF judgement; −, panel disagreed with original IWGDF judgement;?, panel unsure if agreed with original IWGDF judgement due to lack of IWGDF information on judgement; =, panel agreed with original IWGDF judgements during screening (see Table 1); *QoE* Quality of evidence

**Table 3:** Summary of the original IWGDF recommendation compared with the new Australian guideline recommendations for PAD

No	Original IWGDF Recommendation	Decision	New Australian Recommendation
1	Examine the feet of all patients with diabetes annually for the presence of peripheral artery disease even in the absence of foot ulceration. At a minimum, this should include taking a relevant history and palpating foot pulses. (Strong; low)	Adopted	As stated in original IWGDF Recommendation
2	Clinically examine (by relevant history and palpation of foot pulses) all patients with diabetes and foot ulceration for the presence of peripheral artery disease. Clinically examine (by relevant history and palpation of foot pulses) all patients with diabetes and foot ulceration for the presence of PAD. (Strong; low)	Adopted	As stated in original IWGDF Recommendation
3	As clinical examination does not reliably exclude PAD in most persons with diabetes and a foot ulcer, evaluate pedal Doppler arterial waveforms in combination with ankle systolic pressure and systolic ankle brachial index (ABI) or toe systolic pressure and toe brachial index (TBI) measurement. No single modality has been shown to be optimal, and there is no definite threshold value above which PAD can reliably be excluded. However, PAD is a less likely diagnosis in the presence of ABI, 0.9–1.3; TBI, ≥ 0.75; and triphasic pedal Doppler waveforms. (Strong; low)	Adopted	As stated in original IWGDF Recommendation
4	Perform at least one of the following bedside tests in a patient with a diabetes-related foot ulcer and PAD, any of which increases the pretest probability of healing by at least 25%: a skin perfusion pressure of ≥40 mmHg, a toe pressure of≥30 mmHg, or a transcutaneous oxygen pressure (TcPO2) of ≥25 mmHg. (Strong; moderate)	Adopted	As stated in original IWGDF Recommendation
5	Use the Wound, Ischaemia, and foot Infection (WIfI) classification system as a means to stratify amputation risk and revascularisation benefit in a patient with a diabetes-related foot ulcer and PAD. (Strong; moderate)	Adopted	As stated in original IWGDF Recommendation
6	Always consider urgent vascular imaging, and revascularisation, in a patient with a diabetes-related foot ulcer and an ankle pressure of< 50 mmHg, ABI of < 0.5, a toe pressure of < 30 mmHg, or a TcPO2 of < 25 mmHg. (Strong; low)	Adopted	As stated in original IWGDF Recommendation
7	Always consider vascular imaging in patients with a diabetes-related foot ulcer, irrespective of the results of bedside tests, when the ulcer is not healing within 4 to 6 weeks despite good standard of care. (Strong; low)	Adopted	As stated in original IWGDF Recommendation
8	Always consider revascularisation in a patient with a diabetes-related foot ulcer and PAD, irrespective of the results of bedside tests, when the ulcer is not healing within 4 to 6 weeks despite optimal management. (Strong; low)	Adopted	As stated in original IWGDF Recommendation
9	Do not assume diabetes-related microangiopathy, when present, is the cause of poor healing in patients with a diabetes-related foot ulcer; therefore, always consider other possibilities for poor healing. (Strong; low)	Adopted	As stated in original IWGDF Recommendation
10	Use any of the following modalities to obtain anatomical information when considering revascularizing a patient’s lower extremity: colour duplex ultrasound, computed tomographic angiography, magnetic resonance angiography, or intra-arterial digital subtraction angiography. Evaluate the entire lower extremity arterial circulation with detailed visualization of below-the-knee and pedal arteries, in an anteroposterior and lateral plane. (Strong; low)	Adopted	As stated in original IWGDF Recommendation
11	When performing revascularisation in a patient with a diabetes-related foot ulcer, aim to restore direct blood flow to at least one of the foot arteries, preferably the artery that supplies the anatomical region of the ulcer. After the procedure, evaluate its effectiveness with an objective measurement of perfusion. (Strong; low)	Adopted	As stated in original IWGDF Recommendation
12	As evidence is inadequate to establish whether an endovascular, open, or hybrid revascularisation technique is superior, make decisions based on individual factors, such as morphological distribution of PAD, availability of autogenous vein, patient co-morbidities, and local expertise. (Strong; low)	Adopted	As stated in original IWGDF Recommendation
13	Any centre treating patients with a diabetes-related foot ulcer should have expertise in, and rapid access to facilities necessary to diagnose and treat, PAD, including both endovascular techniques and bypass surgery. (Strong; low)	Adapted	Any centre treating patients with a diabetes-related foot ulcer should have expertise in, and/or rapid access to facilities necessary to diagnose and treat, PAD, including both endovascular techniques and bypass surgery. (Strong; low)
14	Ensure that after a revascularisation procedure in a patient with a diabetes-related foot ulcer, the patient is treated by a multidisciplinary team as part of a comprehensive care plan. (Strong; low)	Adopted	As stated in original IWGDF Recommendation
15	Urgently assess and treat patients with signs or symptoms of PAD and a diabetes-related foot infection, as they are at particularly high risk for major limb amputation. (Strong; moderate)	Adopted	As stated in original IWGDF Recommendation
16	Avoid revascularisation in patients in whom, from the patient’s perspective, the risk-benefit ratio for the probability of success of the procedure is unfavourable. (Strong; low)	Adopted	As stated in original IWGDF Recommendation
17	Provide intensive cardiovascular risk management for any patient with diabetes and an ischaemic foot ulcer, including support for cessation of smoking, treatment of hypertension, control of glycaemia, and treatment with a statin drug as well as low-dose clopidogrel or aspirin. (Strong; low)	Adopted	As stated in original IWGDF Recommendation

Underlined wording indicates the specific adapted changes to the original IWGDF recommendation

Two responses to the public consultation survey were received with both responding that they strongly agreed that the guideline should be approved as the new Australian PAD guideline, that the guideline would be supported by the majority of their colleagues and if approved they would encourage its use in practice. All de-identified feedback comments received during public consultation and the panel’s responses to each comment were collated and posted on the Diabetes Feet Australia website. Based on the collated public consultation feedback, the guideline was revised, approved by the panel and Australian DFD Guidelines working group, and endorsed as the new *Australian guideline on diagnosis and management of peripheral artery disease* by nine peak national bodies including the Australian and New Zealand Society for Vascular Surgery, Australian Podiatry Association, Wounds Australia, Australasian Society for Infectious Diseases, Australian Orthotic Prosthetic Association, Pedorthic Association of Australia, Australian Aboriginal and Torres Strait Islander Diabetes-related Foot Complications Program, Australian Diabetes Society and Diabetes Feet Australia.

**Fig. 1:**
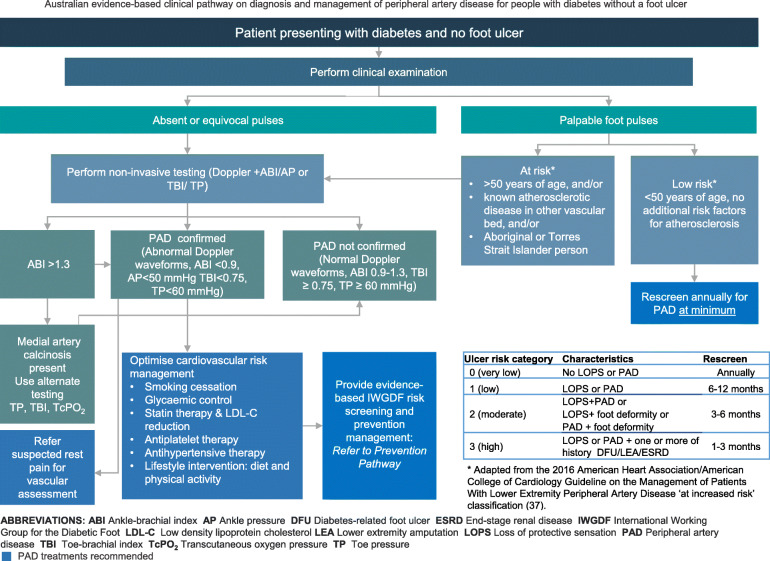
Australian clinical pathway to guide evidence-based diagnosis and management of PAD for people with diabetes without foot ulcers

**Fig. 2:**
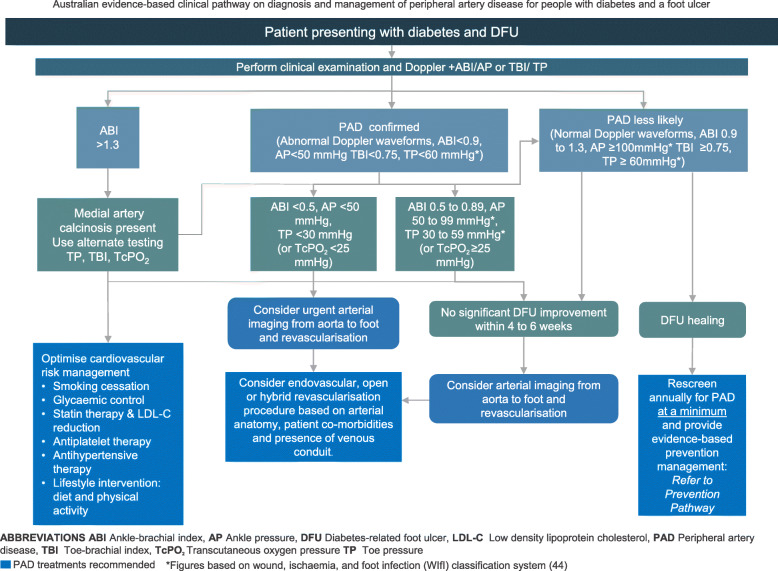
Australian clinical pathway to guide evidence-based diagnosis and management of PAD for people with diabetes and diabetes-related foot ulcers

In the subsequent section each of the 17 Australian PAD recommendation are listed. In addition, the question addressed by the recommendation, the panel decision and rationale to adopt, adapt or exclude; summary justification and detailed justification where applicable for the recommendation; and considerations for implementation including for specific subgroups (including for Aboriginal and Torres Strait Islander and geographically remote populations), summary monitoring and potential future research priorities are provided. For detailed justifications, implementation, monitoring and research considerations for each recommendation see the eTables in the Supplementary Material. Finally, all recommendations are incorporated in two consensus Australian clinical pathways to guide evidence-based diagnosis and management of PAD people with diabetes (Figs. 1 and 2).

The recommendations are displayed in order under the categories of A. Diagnosis, B. Prognosis and C. Treatment. A glossary of definitions is included at the end of the document.

## Diagnosis

### Question one

In a person with diabetes and no foot ulceration, which symptoms and signs (clinical examination) should clinicians examine in order to identify or exclude PAD?

#### Recommendation 1

Examine the feet of all patients with diabetes annually for the presence of peripheral artery disease even in the absence of foot ulceration. At a minimum, this should include taking a relevant history and palpating foot pulses. (Strength of the recommendation: strong; quality of the evidence: low).

#### Decision

Adopted.

#### Rationale

The panel decided to adopt this recommendation. The panel agreed with the judgements of the IWGDF and considered this recommendation to be acceptable and applicable in the Australian context (Table 1).

##### Summary justification

The panel agreed with the IWGDF that the recommendation was strong based on the balance of effects favouring the annual screening over no screening, and that the quality of evidence was low. There are currently limited data investigating the diagnostic accuracy of signs/symptoms or pulse palpation for PAD [19, 36]. The panel agreed with the IWGDF that this recommendation is consistent with current international guidelines where annual screening for PAD is recommended for all people with diabetes [37–39]. The panel agreed this recommendation is compatible with Australian culture and the Australian health care setting, that the necessary expertise is widely available, and there is no limitation on implementation of this recommendation due to lack of equipment, or Australian healthcare legislation or policies. A clinical pathway for diagnosis and management of PAD in people with diabetes without DFU is provided in Fig. 1.

#### Implementation considerations

##### General considerations

In people with diabetes, PAD is frequently asymptomatic, or has atypical symptoms [40, 41]. For example, peripheral neuropathy can mask pain symptoms and autonomic neuropathy can result in a warm foot, meaning that the widely recognised signs and symptoms of PAD may not be present [40–42]. While this recommendation is applicable to all people with diabetes with and without DFU, where there are clinical signs and symptoms of PAD more frequent screening and further vascular imaging may be necessary. Further investigation with bedside testing is also recommended in populations considered at higher risk of PAD including those over 50 years of age, those wiith diagnosed atherosclerosis in another vascular bed and Aboriginal and Torres Strait Islander people [3, 37]. In addition, the high incidence of cardiovascular disease co-existing with PAD necessitates additional cardiovascular risk management in this population to reduce risk of myocardial infarction and stroke [21].

##### Geographically remote people

Given that a range of health professionals have the expertise to conduct a clinical examination for PAD including history taking and pulse palpation, the panel considered that such a service should be available in more geographically remote areas.

##### Aboriginal and Torres Strait Islander people

More frequent screening may be required and further bedside testing in the population should be used due to increased risk of PAD [3]. Basic PAD screening can be provided by a range of health professionals including appropriately trained Aboriginal Health Workers. This may assist in timely screening being provided to Aboriginal and Torres Strait Islander Communities in more geographically remote areas. The panel agreed on the high importance of involving an Aboriginal Health Worker in care delivery. The panel also agreed on the importance of explaining the need for, and nature of, the assessment, and discussing the results with the patient and their family using a professional interpreter when required.

For detailed implementation, monitoring and research considerations see eTable A1 in Supplementary Material.

### Question two

In a person with diabetes and a foot ulcer, which symptoms and signs (clinical examination) should clinicians examine in order to identify or exclude PAD?

#### Recommendation 2

Clinically examine (by relevant history and palpation of foot pulses) all patients with diabetes and foot ulceration for the presence of peripheral artery disease. (Strong; low).

Decision: Adopted.

Rationale: The panel decided to adopt this recommendation. The panel agreed with the judgements of the IWGDF and considered this recommendation to be acceptable and applicable in the Australian context (Table 1).

##### Summary justification

The panel agreed with the IWGDF that the quality of available evidence was low as there are little available data investigating the diagnostic accuracy of the presence of signs and symptoms or pulse palpation for identifying PAD in people with DFU. The panel also agreed with the IWGDF, that the recommendation to examine the feet of all patients with diabetes was strong as most patients and health care providers would place high importance on DFU healing over other outcomes, and that the risk was significantly outweighed by the benefit of the assessment. The panel agreed this recommendation is compatible with Australian culture and the Australian health care setting, that the necessary expertise is widely available, and, there is no limitation on implementation of this recommendation due to lack of equipment or Australian healthcare legislation or policies. A clinical pathway for diagnosis and management of PAD in people with diabetes with DFU is provided in Fig. 2.

#### Implementation considerations

##### General considerations

These are consistent with general considerations for recommendation 1.

##### Geographically remote people

These are consistent with general considerations for recommendation 1.

##### Aboriginal and Torres Strait Islander people

In addition to the considerations detailed in recommendation 1, the panel noted that due to the heightened risk of poor outcomes for DFU in Aboriginal and Torres Strait Islander people, and the increased likelihood of PAD in this population, clinical examination that does not suggest presence of PAD should be treated with an abundance of caution [3]. It is particularly important in this population that further bedside testing is conducted as an adjunct to basic clinical examination.

For detailed implementation, monitoring and research considerations see eTable A2 in Supplementary Material.

### Question three

In a person with diabetes and a foot ulcer which “bedside” diagnostic procedure, alone or in combination, has the best performance in diagnosing or excluding PAD?

#### Recommendation 3

As clinical examination does not reliably exclude PAD in most persons with diabetes and a foot ulcer, evaluate pedal Doppler arterial waveforms in combination with ankle systolic pressure and systolic ankle brachial index (ABI) or toe systolic pressure and toe brachial index (TBI) measurement. No single modality has been shown to be optimal, and there is no definite threshold value above which PAD can reliably be excluded. However, PAD is a less likely diagnosis in the presence of ABI, 0.9–1.3; TBI ≥ 0.75; and triphasic pedal Doppler waveforms. (Strong; low).

Decision: Adopted.

Rationale: The panel decided to adopt this recommendation. The panel agreed with the judgements of the IWGDF and considered this recommendation to be acceptable and applicable in the Australian context (Table 1).

##### Summary justification

The panel agreed with IWGDF that there was a low quality of supporting evidence, with a strong recommendation based on the high likelihood that most patients and care providers would consider the benefits of testing outweigh any risk, and would place critical importance on DFU healing over other outcomes. The panel also agreed that these bedside diagnostic tests are applicable to the Australian context, that there is adequate availability of knowledge and skills in objective lower limb vascular assessment, and, that there are no constraints from current legislation or policy to prevent implementation in Australia.

#### Implementation considerations

##### General considerations

A range of health professionals are able to undertake bedside diagnostic vascular testing of the lower limb. Provision of appropriate equipment and training to health professionals caring for people with DFU is necessary to ensure adequate testing can be conducted. Of note, there is not enough evidence to determine if there is any single, or combination of bedside tests, which has greater diagnostic accuracy for PAD. Therefore choice of test or tests should be made based on available equipment and expertise at any given location, and in consideration of limitations of the capacity of each of these tests to accurately identify the presence of significant PAD.

##### Geographically remote people

There may be restricted access to appropriate expertise and equipment in geographically remote areas. However the panel felt that where there are existing health services providing DFU treatment and management, the required bedside testing should be available with choice of test or tests directed by availability of specific equipment and expertise.

##### Aboriginal and Torres Strait Islander people

This recommendation is applicable to Aboriginal and Torres Strait Islander people. Considerations for this recommendation are consistent with those provided in Recommendations 1 and 2.

For detailed implementation, monitoring and research considerations see eTable B3 in Supplementary Material.

## Prognosis

### Question four (recommendations 4–9)

In a person with diabetes, foot ulceration and PAD, which clinical signs, symptoms or non-invasive bedside tests may predict ulcer healing and amputation?

#### Recommendation 4

Perform at least one of the following bedside tests in a patient with a diabetes-related foot ulcer and PAD, any of which increases the pretest probability of healing by at least 25%: a skin perfusion pressure of ≥40 mmHg, a toe pressure of ≥30 mmHg, or a transcutaneous oxygen pressure (TcPO_2_) of ≥25 mmHg. (Strong; moderate).

Decision: Adopted.

Rationale: The panel decided to adopt this recommendation. The panel agreed with the judgements of the IWGDF and considered this recommendation to be acceptable and applicable in the Australian context (Table 1).

##### Summary justification

The panel agreed with the IWGDF recommendation of the quality of supporting evidence (moderate), with a strong recommendation as most patients would place high importance on ulcer healing over other outcomes, and that the risk was significantly outweighed by the benefit of the assessment.

The panel was in agreement that the intervention is applicable to the Australian context, that there is adequate availability of knowledge and skills to implement the above testing methods, and, that expertise and equipment would be available in the majority of health care settings providing such patient services. The panel also agreed that there are no constraints from current legislation or policy.

Implementation considerations:

##### General considerations

In a small number of studies (although outcomes are variable) there is evidence that skin perfusion pressure of ≥40 mmHg, toe pressure ≥ 30 mmHg, or TcPO_2_ ≥ 25 mmHg have individually been shown to increase the probability of DFU healing by more than 25% [31]. The panel agreed that these findings suggest the above thresholds are useful in determining patient suitability for initial implementation of conservative therapy prior to considering revascularisation. This is on the provision that the results of assessment of peripheral perfusion are considered in the context of the presence or absence of other factors, for example infection, which may further impede healing. In addition, in circumstances where there are pressures above these bedside testing thresholds, due to limitations in all the diagnostic testing methods recommended (TcPO_2_, skin perfusion pressure and toe pressures), and the lack of consistency in their accuracy for predicting healing in the literature, PAD should not be excluded as a contributor to poor wound healing when there is a lack of response to optimal care [21]. Similarly, where there are other factors indicating poor healing prognosis including presence of extensive infection or large wound surface area, urgent imaging and potential revascularisation should still be considered [43].

##### Geographically remote people

Lack of specialised equipment, particularly for measuring skin perfusion pressure and TcPO_2_, may limit choice of testing being conducted in remote areas. However as health care centres treating people with DFU in remote areas should routinely be performing bedside testing for PAD in patients, toe pressures are a suitable measure.

##### Aboriginal and Torres Strait Islander people

This recommendation is applicable to Aboriginal and Torres Strait Islander people. Considerations for this recommendation in this population are consistent with those detailed in recommendations 1 and 2. In addition, the panel noted the need to consider the results of the vascular testing performed within the context of other risk factors for non-healing in the population is particularly important. To the panel’s knowledge there are currently no data investigating the capacity for skin perfusion pressure, TcPO_2_ or toe pressure to predict likelihood of DFU healing specifically in Aboriginal and Torres Strait Islander people.

For detailed implementation, monitoring and research considerations see eTable B4 in Supplementary Material.

#### Recommendation 5

Use the Wound, Ischaemia, and foot Infection (WIfI) classification system as a means to stratify amputation risk and revascularisation benefit in a patient with a diabetes-related foot ulcer and PAD. (Strong; moderate).

Decision: Adopted.

Rationale: The panel decided to adopt this recommendation. The panel agreed with the judgements of the IWGDF and considered this recommendation to be acceptable and applicable in the Australian context (Table 1).

##### Summary justification

The WIfI classification provides a guide to estimate risk of amputation and potential benefit of revascularisation based on the ulcer, severity of ischaemia measured via non-invasive bedside testing, and infection severity (using IWGDF/Infectious Diseases Society of America classification) [44]. The panel was in agreement with the IWGDF with a strong recommendation, with the balance of effects for patients and health care providers strongly favouring use of the WIfI system. The panel also agreed with the moderate rating for the quality of available evidence with the WIfI system validated for use in people with diabetes [44, 45]. The panel also agreed with the IWGDF that the application of the WIfI classification system would be acceptable for the majority of Australian patients, and would be applicable as there are no legislative or policy constraints on its use. The classification system is readily available and can be downloaded as a calculator tool to assist with application [46].

Implementation considerations

##### General considerations

Given the availability of the WIfI tool, and its use of non-invasive bedside testing to determine level of ischaemia, and clinical grading of infection and the wound, the panel agreed there would be no specific limitations to implementation.

##### Geographically remote people

The panel agreed that the nature of the classification system, including use of bedside testing and clinical grading of wound and infection severity makes it suitable for use in geographically isolated areas.

##### Aboriginal and Torres Strait Islander people

The panel agreed that this recommendation is generally applicable to Aboriginal and Torres Strait Islander people. As the WIfI tool has not been validated in this specific population, as per recommendation 4, the disproportionately high risk of amputation in Aboriginal and Torres Strait Islander people particularly in rural and remote areas, and extrinsic and cultural barriers to care access (for example the need to stay on Country, family and Community circumstances and roles, and the preference for Community-delivered care) need to be considered in addition to the WIfI classification system to better determine risk of amputation and benefits of revascularisation.

For detailed implementation, monitoring and research considerations see eTable B5 in Supplementary Material.

#### Recommendation 6

Always consider urgent vascular imaging, and revascularisation, in a patient with a diabetes-related foot ulcer and an ankle pressure of < 50 mmHg, ABI of < 0.5, a toe pressure of < 30 mmHg, or a TcPO_2_ of < 25 mmHg. (Strong; low).

Decision: Adopted.

Rationale: The panel decided to adopt this recommendation. The panel agreed with the judgements of the IWGDF and considered this recommendation to be acceptable and applicable in the Australian context (Table 1).

##### Summary justification

The panel agreed with the IWGDF that the recommendation was strong, with low quality of supporting evidence. In addition, the panel agreed that there would probably be no important uncertainty in relation to the majority of Australian patients preferring imaging and consideration of urgent revascularisation compared to no intervention where likelihood of successful healing with conservative care is very low. The panel also concluded that the recommendation is applicable, that there is no legislative or policy constraints to its use, and, that the resources and expertise are available for the majority of patients and health care providers in health care settings typically providing such treatment services in Australia.

#### Implementation considerations

##### General considerations

These are consistent with the general considerations for recommendation 5.

##### Geographically remote people

This recommendation is applicable to geographically remote locations, however in these situations timely referral for imaging and revascularisation require well established rapid referral pathways which should be developed in consideration of the local availability of services and expertise.

##### Aboriginal and Torres Strait Islander people

This recommendation is applicable to Aboriginal and Torres Strait Islander people. The reader is referred to considerations in recommendation 1 and 2. The panel also agreed on the importance of explaining the need for, and nature of, any further vascular intervention or surgical intervention including the expected timeframes for, and location of, related hospitalisation and longer term post-operative care with the patient and their family using a professional interpreter when required. Furthermore due to the disproportionately high risk of amputation in Aboriginal and Torres Strait Islander people particularly in rural and remote areas, extrinsic and cultural barriers to care access need to be considered in the establishment of appropriate rapid referral pathways and in considering revascularisation procedures.

For detailed implementation, monitoring and research considerations see eTable B6 in Supplementary Material.

#### Recommendation 7

Always consider vascular imaging in patients with a diabetes-related foot ulcer, irrespective of the results of bedside tests, when the ulcer is not healing within 4 to 6 weeks despite good standard of care. (Strong; low).

Decision Adopted.

Rationale: The panel decided to adopt this recommendation. The panel agreed with the judgements of the IWGDF and considered this recommendation to be acceptable and applicable in the Australian context (Table 1).

##### Summary justification

The panel was in agreement with the IWGDF in regard to both the strength of the recommendation (strong) and the quality of available evidence (low). In addition, the panel agreed that there would be no important uncertainty in relation to the majority of Australian patients preferring the intervention (imaging) and valuing DFU healing over other outcomes. The panel considered that this recommendation was applicable to the Australian context, that there were no policy or legislative constraints on implementation of this recommendation, and, that there is adequate expertise and equipment available in secondary and tertiary health care settings were patients typically access this care.

#### Implementation considerations

##### General considerations

As discussed in recommendation 3, the paucity of available research investigating diagnostic accuracy of bedside testing for PAD in patients with DFU highlights the limited capacity for this testing to rule out presence of the disease. Undiagnosed ischaemia is therefore a potential contributing factor to delayed healing in situations where appropriate conservative care is being provided. Current available evidence suggests the timeframe for implementing additional vascular imaging and undertaking revascularisation where appropriate influences healing outcomes [47].

##### Geographically remote people

Differing levels of accessibility to conservative DFU care in remote regions may affect ulcer healing outcomes including time to achieve healing. A good standard of multidisciplinary DFU care involves regular debridement and wound dressing, as well as effective pressure offloading and rapid control of the presence of infection. In more geographically remote areas, delays or more extended time between appointments, as well as hot or dry and dusty environments, may reduce adherence to some conservative therapies (for example, offloading devices). This may also slow the healing time. Nevertheless, due to the need to diagnose PAD as soon as possible where delayed healing is occurring further imaging should be sought.

##### Aboriginal and Torres Strait Islander people

This recommendation is applicable to Aboriginal and Torres Strait Islander people. The reader is referred to considerations in recommendation 1 and 2 and to the considerations for those living in geographically remote areas as described above, and additional considerations for Aboriginal and Torres Strait Islander people in recommendation 6. In addition, the panel agreed that there may be the need to remove protective offloading devices for Community-based cultural activities and this should be reflected in individual management plans with adjustments to monitoring where required. Due to the high incidence of PAD in Aboriginal and Torres Strait Islander people, further imaging should be sought where there is practitioner concern over healing response.

For detailed implementation, monitoring and research considerations see eTable B7 in Supplementary Material.

#### Recommendation 8

Always consider revascularisation in a patient with a diabetes-related foot ulcer and PAD, irrespective of the results of bedside tests, when the ulcer is not healing within 4 to 6 weeks despite optimal management. (Strong; low).

Decision Adopted.

Rationale: The panel decided to adopt this recommendation. The panel agreed with the judgements of the IWGDF and considered this recommendation to be acceptable and applicable in the Australian context (Table 1).

##### Summary justification

That panel was in agreement with the IWGDF on the strength of this recommendation (strong) and the low level of available evidence. The panel agreed that the intervention is acceptable and feasible in the Australian context with the majority of Australian patients preferring revascularisation and valuing DFU healing over other outcomes. The panel agreed that there were no policy or legislative constraints on implementation of this recommendation, and that there is adequate expertise and equipment available in health care settings where patients typically access this care. The panel noted applicability of the recommendation is likely to vary between patients as DFU are frequently complex with multiple contributing factors including infection, ischaemia and neuropathy. Determining the most appropriate trial duration for conservative care is therefore challenging and likely to vary between individuals.

#### Implementation considerations

##### General considerations

These are consistent with general considerations for recommendation 7.

##### Geographically remote people

As per recommendation 7, difficulties for patients regularly accessing optimal conservative care either due to distance or service availability may contribute to delayed healing. This highlights the need for individual patient circumstances and results of vascular imaging to be used to inform decisions relating to revascularisation.

##### Aboriginal and Torres Strait Islander people

This recommendation is applicable to Aboriginal and Torres Strait Islander people. Specific considerations for this recommendation are consistent with those outlined in recommendations 1, 2 and 6 in relation to care delivery involving an Aboriginal Health Worker, and the need for effective patient and family communication regarding assessment and treatment options. As per recommendation 7, the panel noted Aboriginal and Torres Strait Islander people may experience reduced frequency of access to appropriate care due to cultural barriers and lack of culturally safe care, as well as difficulty due to geographical remoteness. This may have an adverse effect on healing rates, and, as with those living in remote geographical areas, such circumstances should be considered in addition to vascular imaging when contemplating revascularisation.

For detailed implementation, monitoring and research considerations see eTable B8 in Supplementary Material.

#### Recommendation 9

Do not assume diabetes-related microangiopathy, when present, is the cause of poor healing in patients with a diabetes-related foot ulcer; therefore, always consider other possibilities for poor healing. (Strong; low).

Decision: Adopted.

Rationale: The panel decided to adopt this recommendation. The panel agreed with the judgements of the IWGDF and considered this recommendation to be acceptable and applicable in the Australian context (Table 1).

##### Summary justification

The panel agreed with IWGDF that there was a low quality of supporting evidence, with a strong recommendation based on expert opinion. The panel also agreed that this recommendation is acceptable to the Australian setting, and, that there would be no important uncertainty in relation to the majority of Australian patients preferring all likely causes of poor healing to be investigated. The panel agreed that the intervention is applicable to the Australian context, that there is adequate availability of knowledge and skills in assessment of the foot with diabetes, and that there are no policy or legislative constraints on implementation of this recommendation.

#### Implementation considerations

##### General considerations

Diabetes-related microangiopathy is characterised by increased capillary basement membrane thickening and is proposed to have a deleterious effect on wound healing. Presence of neuropathy is proposed to further contribute to localised tissue hypoxia and reduced vasodilatory capacity of the microvasculature in response to injury [48, 49]. However, due to the lack of compelling evidence supporting a role of microangiopathy in poor DFU healing, the panel agreed with the IWGDF that other factors that may impair wound healing and reduce peripheral perfusion including PAD undiagnosed by bedside testing, presence of high plantar pressures, oedema and infection should be considered first and foremost.

##### Geographically remote people

The panel consider this recommendation to be applicable to people living in geographically remote areas. The panel noted the importance of thorough investigation of both intrinsic (e.g. infection, PAD) and extrinsic (e.g. access to care) factors for delayed healing in geographically remote people.

##### Aboriginal and Torres Strait Islander people

The panel considered this recommendation to be suitable for Aboriginal and Torres Strait Islander people, but, as per recommendations 6 and 7, identified the need to consider extrinsic factors that may contribute to delayed, or non-healing in this population. These include adequate access to culturally safe care, suitability of conservative care to cultural needs, and similar potential restrictions in access to regular conservative care in geographically remote areas.

For detailed implementation, monitoring and research considerations see eTable B9 in Supplementary Material.

## Treatment

### Question five (recommendation 10)

In a person with diabetes and foot ulceration, which diagnostic imaging modalities to obtain anatomical information are most useful when considering revascularisation?

#### Recommendation 10

Use any of the following modalities to obtain anatomical information when considering revascularising a patient’s lower extremity: colour duplex ultrasound (CDUS), computed tomographic angiography (CTA), magnetic resonance angiography (MRA), or intra-arterial digital subtraction angiography (DSA). Evaluate the entire lower extremity arterial circulation with detailed visualisation of below-the-knee and pedal arteries, in an anteroposterior and lateral plane. (Strong; low).

Decision: Adopted.

Rationale: The panel decided to adopt this recommendation. The panel agreed with the judgements of the IWGDF and considered this recommendation to be acceptable and applicable in the Australian context (Table 1).

##### Summary justification

The panel agreed with the IWGDF judgement on the strength of the recommendation (strong), with low quality of available evidence. Revascularisation of the lower limb should be guided by appropriate imaging of the entire lower limb arterial circulation including pedal circulation. Detailed visualisation of vessels below the knee and the pedal arteries is required due to increased likelihood of distally located disease in people with diabetes [15]. CDUS, MRA, CTA and DSA are all modalities that may be used to establish lower limb circulation in a patient with diabetes. The panel agreed the majority of Australian patients would prefer to undergo imaging. The panel agreed that the intervention is applicable to the Australian context and that there were no policy or legislative constraints on implementation of this recommendation. The panel noted that choice of imaging may be influenced by the availability of expertise and equipment, and patient specific factors (see below: implementation considerations), however, the panel considered there to be adequate expertise and equipment available in secondary and tertiary health care settings where patients typically access this care.

#### Implementation considerations

##### General considerations

The panel agreed with the IWGDF that CDUS, CTA, MRA, or DSA could be used for evaluation of lower limb arterial circulation. Each form of imaging has specific limitations and contraindications which need to be considered in the selection of the type of imaging used. In brief, presence of significant calcification reduces the accuracy of CDUS and CTA. Multi-segment disease and oedema also reduce the imaging capability of CDUS. Imaging requiring contrast agents including MRA, CTA, and DSA are contraindicated where there is allergy to the contrast agent or there is significant risk of nephrotoxicity. MRA is also contraindicated in those patients with cardiac pacemakers, and some other implants and in claustrophobic patients without sedation.

##### Geographically remote people

The panel agreed that while a range of imaging services may be available in metropolitan and regional areas, this access is likely to be very limited in geographically remote areas. In such situations the importance of well-established clinical referral pathways to support timely access to services is paramount.

##### Aboriginal and Torres Strait Islander people

The panel considered that this recommendation was appropriate for Aboriginal and Torres Strait Islander people. Consistent with populations in remote geographical areas, the importance of established referral pathways developed in conjunction with Community-based Aboriginal Health and Medical Services and where the care provision is supported by an Aboriginal Health Worker, is integral to optimising patient outcomes. In addition, the reader is referred to considerations for Aboriginal and Torres Strait Islander people for recommendations 1 and 2.

For detailed implementation, monitoring and research considerations see eTable C10 in Supplementary Material.

### Question six (recommendations 11–15)

What are the aims and methods of revascularisation and onward management in a person with diabetes, foot ulceration, and PAD?

#### Recommendation 11

When performing revascularisation in a patient with a diabetes-related foot ulcer, aim to restore direct blood flow to at least one of the foot arteries, preferably the artery that supplies the anatomical region of the ulcer. After the procedure, evaluate its effectiveness with an objective measurement of perfusion. (Strong; low).

Decision: Adopted.

Rationale: The panel decided to adopt this recommendation. The panel agreed with the judgements of the IWGDF and considered this recommendation to be acceptable and applicable in the Australian context (Table 1).

##### Summary justification

The panel was in agreement with the IWGDF regarding the strength of the recommendation (strong) based on the balance of effects favouring revascularisation over no intervention for improving tissue perfusion and DFU healing. The panel also agreed with the IWGDF on the quality of the available evidence (low) due to lack of reporting of included study populations, inconsistent application of interventions and the poor control of potential confounders.

The panel agreed that the intervention is applicable to the Australian context with the majority of Australian patients preferring revascularisation and valuing DFU healing and limb salvage over other outcomes. The panel also agreed that there were no policy or legislative constraints on implementation of this recommendation, and that there is adequate expertise and equipment available in health care settings where patients typically access this care.

#### Implementation considerations

##### General considerations

While the most effective approach to revascularisation remains a point of contention, the panel agreed with the IWGDF that direct revascularisation, where there is restoration of flow to the anatomical area in which the ulcer is located, will theoretically be more effective than an indirect technique. The panel also agreed that in the presence of end-stage renal disease revascularisation needs to be carefully considered due to high rates of complications, a 5 year mortality rate of up to 91% and moderate limb salvages rates (65–70%) for those surviving to 1 year [21]. The panel agreed with the IWGDF that, in the presence of extensive infection, therapy should be implemented to control the infection prior to undertaking a revascularisation procedure and subsequent restoration of perfusion should be undertaken within a few days of stabilisation of the patient [21].

##### Geographically remote people

The panel agreed that this recommendation is applicable to people living in geographically remote areas. The panel noted that, for these patients, rapid referral pathways are required to treatment centres offering revascularisation procedures and that access to appropriate follow-up assessments and care needs to be established as part of the management model in conjunction with involved health care providers. Options to support health practitioners in remote areas with appropriate expertise via telehealth and other forms of remote monitoring should be considered.

##### Aboriginal and Torres Strait Islander people

The panel considered this recommendation to be applicable to Aboriginal and Torres Strait Islander people. Consistent with recommendation 6, the panel agreed on the importance of explaining the need for, and nature of, any further vascular intervention or surgical intervention including the expected timeframes for, and location of, related hospitalisation and longer-term post-operative care with the patient and their family using a professional interpreter when required. Furthermore, established referral pathways, as well as appropriate, culturally safe follow-up care, are required for Aboriginal and Torres Strait Islander people in all geographical locations. These should be developed in conjunction with Community-based Aboriginal Health and Medical Services where the care access and provision is supported by an Aboriginal Health Worker and professional interpreter (where required) to optimise patient outcomes.

For detailed implementation, monitoring and research considerations see eTable C11 in Supplementary Material.

#### Recommendation 12

As evidence is inadequate to establish whether an endovascular, open, or hybrid revascularisation technique is superior, make decisions based on individual factors, such as morphological distribution of PAD, availability of autogenous vein, patient co-morbidities, and local expertise. (Strong; low).

Decision: Adopted.

Rationale: The panel decided to adopt this recommendation. The panel agreed with the judgements of the IWGDF and considered this recommendation to be acceptable and applicable in the Australian context (Table 1).

##### Summary justification

Review of the literature reporting DFU healing and limb salvage outcomes following endovascular and open techniques show these to be similar. However there is a lack of comparative studies evaluating endovascular, open or hybrid techniques in people with diabetes. The panel therefore agreed with the IWGDF on the strength of recommendation (strong) based on a low level of quality of available evidence, and the need for centres treating people with DFU to be able to provide a range of surgical treatment options.

The panel agreed that there would probably be no important uncertainty in relation to the majority of Australian patients preferring the intervention and valuing DFU healing over other outcomes. The panel considered that this recommendation was applicable to the Australian context, that there are no policy or legislative constraints on implementation of this recommendation, and, that there is adequate expertise and equipment available in health care settings where patients typically access this care.

#### Implementation considerations

##### General considerations

The panel agreed with the IWGDF that the complex nature of diabetes-related PAD, supports the patient-specific approach to selection of revascularisation techniques.

##### Geographically remote people

This recommendation is applicable to people in geographically remote areas, however, the panel agreed that access to expertise may be variable in some locations and that considerations for this subgroup are consistent with those for recommendation 11.

##### Aboriginal and Torres Strait Islander people

The panel considered that this recommendation was appropriate for Aboriginal and Torres Strait Islander people and considerations for this subgroup are the same as for recommendation 11.

For detailed implementation, monitoring and research considerations see eTable C12 in Supplementary Material.

#### Recommendation 13

Any centre treating patients with a diabetes-related foot ulcer should have expertise in, and/or rapid access to facilities necessary to diagnose and treat, PAD, including both endovascular techniques and bypass surgery. (Strong; low).

Decision: Adapted.

Rationale: The panel agreed with the judgements of the IWGDF in relation to the acceptability of the recommendation. The panel decided to adapt this recommendation based on the panel having a difference in judgement of the applicability, specifically in relation to the feasibility of the recommendation in the Australian context (Table 1). Therefore the wording changes to original IWGDF included the addition of ‘and/or’.

##### Summary justification

The panel agreed with the strength of the recommendation (strong) and the low quality of the available evidence. As per recommendation 12, the panel noted the complex nature of patients presenting with PAD and DFU requiring the availability of a range of surgical treatment options. The panel also agreed that the need for urgent medical intervention particularly in the presence of infection, as well as the short optimal timeframe for revascularisation supports the need for rapid access to diagnostic and treatment services.

The panel agreed that there would probably be no important uncertainty in relation to the majority of Australian patients preferring the intervention and valuing DFU healing over other outcomes. The panel were unsure that having expertise in, and rapid access to, facilities necessary to diagnose and treat PAD including both endovascular techniques and bypass surgery in any centre treating DFU was feasible in the Australian context due to the geographical isolation of many parts of the country. The detailed justifications from our full assessment are provided below.

#### Detailed justifications

Problem: PAD is estimated to be present in up to 50% of DFU and to be an independent risk factor in their development [10, 11]. The panel agreed that DFU and ischaemia are associated with increased risk of amputation and delay in revascularisation is associated with poorer outcomes. This supports the need for centres treating DFU to have expertise in non-invasive diagnosis of PAD and, at minimum, rapid access to facilities necessary to treat PAD including access to both endovascular and bypass surgery.

##### Desirable effects

The panel agreed with the IWGDF that that there was a large anticipated benefit of revascularisation over conservative care based on a limb salvage rate at 1 year of 82% following revascularisation versus 50–54% in patients deemed unsuitable for revascularisation and receiving conservative care [21].

##### Undesirable effects

The panel agreed with the IWGDF that the available evidence supported that the difference in undesirable effects associated with revascularisation was small. This was based on the available evidence showing improved healing and limb salvage outcomes at 1 year following revascularization. Specifically, higher amputation rates (approximately 50%) associated with conservative care in those with DFU and ischaemia at 1 year follow up have been demonstrated compared to those undergoing revascularisation (approximately 18%) at 1 year follow up [21, 50, 51].

##### Quality (or certainty) of evidence

The panel agreed with the IWGDF that the quality of evidence was low. This was based on observational and restrospective data demonstrating shorter time periods to revascularisation of between 2 and 8 weeks were associated with higher probability of DFU healing and lower likelihood of limb loss [47, 52].

##### Values

The panel agreed with the IWGDF that there was probably no important uncertainty or variability in the extent to which patients valued the outcome measures used to compare the intervention (revascularisation) versus conservative care, such as healing and amputation.

##### Balance of effects

Although there is a low level of evidence, the panel agreed with the IWGDF that the recommendation was strong based on large desirable effects on healing outcomes and limb salvage rates and trivial undesirable effects on adverse events with vascular intervention in patients with ischaemic DFU.

##### Acceptability

The panel agreed with the IWGDF that revascularisation with either endovascular techniques and/or bypass surgery would be acceptable to the majority of patients and providers in most healthcare settings that typically provide such services in Australia. This was on the basis that the panel considered that most Australian patients and providers would accept the evidence that the balance of effects was in favour of revascularisation over conservative care in the presence of DFU with ischaemia.

##### Feasiblity

The panel members were unsure if they agreed with the IWGDF on the feasibility of this recommendation in the Australian context. The basis of the uncertainty related to the recommendation that all centres treating DFU have expertise in, and rapid access to facilities necessary to diagnose and treat, PAD, including both endovascular techniques and bypass surgery. The expert opinion of the panel was that such expertise and facilities were not available at all centres treating DFU in Australia. The panel recognised that high service costs and low target populations challenge viability of health care provision in regional and remote areas, and, that this applied to the specialised services and facilities required for advanced diagnosis and surgical interventions for PAD. The panel agreed that in these circumstances, in addition to ensuring availability of appropriate bedside vascular testing onsite, establishing formal pathways to ensure rapid access to such facilities and expertise was appropriate for centres treating DFU in regional and rural Australia.

#### Implementation considerations

##### General considerations

The panel agreed with the IWGDF regarding the need for rapid access to further vascular imaging and revascularisation services based on evidence of improved outcomes with prompt revascularisation intervention [47, 52, 53]. Given the lack of evidence to support one form of revascularisation technique over others (i.e. open versus endovascular), the panel agreed with the IWGDF that both techniques should be available [53]. As per recommendation 12, given the complex, multi-system nature of diabetes and the specific complications this causes the panel agreed the patient-specific approach to choice of revascularisation technique is appropriate. Due to the variable nature of the extent of health care services available throughout rural and regional Australia and, related to this, the differing availability of services to provide post-operative follow-up care, the panel noted the need for development of local pathways specific to the needs of individual DFU centres. The panel also identified that, as per recommendations 11 and 12, telehealth and other forms of remote monitoring provide mechanisms to support health practitioners, referral pathways and care models in rural and remote areas. Facilitation of rapid referral and provision of appropriate expertise via these mechanisms should be integrated into the development of local referral pathways, and as part of the management model in conjunction with involved health care providers. As alternatives to providing onsite care in geographical regions with small populations, the panel agreed these resources should be prioritised for future government and health services funding to support a nation-wide approach to provision of optimal DFU care.

##### Geographically remote people

The reader is referred to the panel’s advice for recommendations 11 and 12.

##### Aboriginal and Torres Strait Islander people

In terms of considerations for Aboriginal and Torres Strait Islander people, the panel’s advice is consistent with recommendation 11.

#### Monitoring considerations

The panel agreed formal monitoring systems to be able to collect, monitor and analyse revascularisation and DFU healing outcomes in accordance with national based High Risk Foot Service database monitoring systems and datasets where applicable to this recommendation [54–56]. This is particularly important to monitor outcomes for patients being referred from rural and remote areas, to include effectiveness of referral processes and wait times.

#### Future research considerations

Existing data demonstrates health disparities for all Australians living in rural and remote areas [57]. Further prospective research assessing comparative outcomes for patients with DFU in rural and regional Australia is required to better inform service delivery models to support patients in these areas. In addition, increasing availability of health technology offers the opportunity to investigate methods to improve access to diabetes-related foot care for people living in rural and remote areas through remote monitoring programs supported by local community health workers, and should be a focus for populations where access to care is restricted and there is high risk of amputation. This is particularly relevant to Aboriginal and Torres Strait Islander Communities with Aboriginal and Torres Strait Islander people comprising up to 91% of those undergoing amputation in rural and remote Australia [20, 58, 59].

#### Recommendation 14

Ensure that after a revascularisation procedure in a patient with a diabetes-related foot ulcer, the patient is treated by a multidisciplinary team as part of a comprehensive care plan. (Strong; low).

Decision: Adopted.

Rationale: The panel decided to adopt this recommendation. The panel agreed with the judgements of the IWGDF and considered this recommendation to be acceptable and applicable in the Australian context (Table 1).

##### Summary justification

The panel concurred with the IWGDF on the strength of this recommendation (strong) and the low quality of available evidence. The panel agreed that the intervention is applicable to the Australian context with the majority of Australian patients preferring DFU healing through use of patient-specific multidisciplinary management over other outcomes. The panel agreed that there were no policy or legislative constraints for implementation of this recommendation, and, that there is adequate expertise and equipment available in health care settings in the majority of locations where patients typically access this care.

#### Implementation considerations

##### General considerations

The IWGDF Practical guidelines on prevention and management of diabetes-related foot disease reflect the multifaceted nature of DFU development and management, and highlight that the restoration of perfusion is only one aspect of a good standard of DFU care [25]. Other aspects of care should include effective pressure offloading and protection of the ulcer, ongoing wound debridement, appropriate management of infection, glycaemic control, and other comorbidities, and patient education, remain essential components of successful management [60].

##### Geographically remote people

The panel agreed that this recommendation was applicable to geographically remote people and the panel’s advice is consistent with recommendations 11 and 12.

##### Aboriginal and Torres Strait Islander people

The panel agreed that this recommendation was applicable to Aboriginal and Torres Strait Islander people and refer the reader to considerations noted for this subgroup in recommendation 11.

For detailed implementation, monitoring and research considerations see eTable C14 in Supplementary Material.

#### Recommendation 15

Urgently assess and treat patients with signs or symptoms of PAD and a diabetes-related foot infection, as they are at particularly high risk for major limb amputation. (Strong; moderate).

Decision: Adopted.

Rationale: The panel decided to adopt this recommendation. The panel agreed with the judgements of the IWGDF and considered this recommendation to be acceptable and applicable in the Australian context (Table 1).

##### Summary justification

The panel was in agreement with the IWGDF that this was a strong recommendation with moderate quality of available evidence. There is a limb loss rate of up to 44% at 12 months for patients with diabetes and foot infection [11]. In Australia, in patients with diabetes-related foot infections, Aboriginal and Torres Strait Islander people have been shown to have a four to six-fold increase in risk of amputation compared to non-Indigenous patients [61]. The panel agreed with the IWGDF that revascularisation should take place promptly following control of significant infection and patient stabilisation and that any further procedures required to restore foot function should be considered after successful revascularisation. The panel agreed that the intervention is applicable to the Australian context with the majority of Australian patients preferring DFU healing and reduction in risk of limb loss. The panel agreed that there were no policy or legislative constraints on implementation of this recommendation. The panel also agreed that there is adequate expertise and equipment available in health care settings in the majority of locations where patients typically access this care.

#### Implementation considerations

##### General considerations

The panel agreed with the IWGDF that revascularisation should take place promptly following control of significant infection and patient stabilisation and that any further procedures required to restore foot function should be considered after successful revascularisation.

##### Geographically remote people

In terms of considerations to use this recommendation in geographically remote people, the panel’s advice is consistent with recommendations 11 and 12.

##### Aboriginal and Torres Strait Islander people

In terms of considerations for Aboriginal and Torres Strait Islander people, the panel’s advice is consistent with recommendation 11.

For detailed implementation, monitoring and research considerations see eTable C15 in Supplementary Material.

### Question seven (recommendation 16)

In a patient with a diabetes-related foot ulcer and PAD, are there any circumstances in which revascularisation should not be performed?

#### Recommendation 16

Avoid revascularisation in patients in whom, from the patient’s perspective, the risk-benefit ratio for the probability of success of the procedure is unfavourable. (Strong; low).

Decision: Adopted.

Rationale: The panel decided to adopt this recommendation. The panel agreed with the judgements of the IWGDF and considered this recommendation to be acceptable and applicable in the Australian context (Table 1).

##### Summary justification

The panel agreed with the IWGDF on the strength of the recommendation (strong) and the low quality of available evidence. The panel also agreed with the IWGDF that, from a patient perspective, a revascularisation procedure may represent an unacceptable risk due to the heightened possibility of perioperative mortality, or due to a limited chance of a favourable surgical outcome.

The panel also agreed that this recommendation is applicable to the Australian context, with the majority of Australian patients preferring avoidance of revascularisation where the risk: benefit ratio is likely to be unfavourable over other management outcomes. The panel agreed that there were no policy or legislative constraints on implementation of this recommendation in Australia. The panel also agreed that there is adequate expertise and equipment in health care settings where the majority of patients typically access DFU care to support implementation of this recommendation.

#### Implementation considerations

##### General considerations

The panel agreed with the IWGDF that a decision to choose conservative care over revascularisation should be discussed with the patient in conjunction with a multidisciplinary care team including a vascular surgeon. Evidence of a 50% healing rate for ischaemic DFU in patients with diabetes unsuitable for revascularisation should also be considered in determining choice of care [50, 51]. Further to this, the panel agreed with the IWGDF that, where a patient was considered to be unsuitable for revascularisation, other experimental techniques including venous arterialisation or intermittent pneumatic compression therapy may offer potential alternative treatments, although their effectiveness has not yet been substantiated.

##### Geographically remote people

The panel agreed that this recommendation was applicable to people in geographically remote locations. Ensuring ease of access to regular ongoing care in the case of conservative treatment should be a priority when developing individual management plans. Use of remote support via telehealth to support local delivery of care both post revascularisation and in patients that are unsuitable for revascularisation should be considered in areas where there are limited local health services.

##### Aboriginal and Torres Strait Islander people

The panel agreed this recommendation was applicable to Aboriginal and Torres Strait Islander people. The panel agreed involvement of Aboriginal and Torres Strait Islander Health Workers and Aboriginal Health and Medical Services and health care providers in discussions relating to vascular intervention and conservative care and subsequent care provision is essential for optimising patient outcomes.

For detailed implementation, monitoring and research considerations see eTable C16 in Supplementary Material.

### Question eight (recommendation 17)

In patients with diabetes, foot ulceration, and PAD, is it possible to reduce the risk of future cardiovascular events?

Provide intensive cardiovascular risk management for any patient with diabetes and an ischaemic foot ulcer, including support for cessation of smoking, treatment of hypertension, control of glycaemia, and treatment with a statin drug as well as low-dose clopidogrel or aspirin. (Strong; low)

Decision: Adopted

Rationale: The panel decided to adopt this recommendation. The panel agreed with the judgements of the IWGDF and considered this recommendation to be acceptable and applicable in the Australian context (Table 1).

#### Summary justification

The panel concurred with the IWGDF on the strength (strong) of this recommedation and the low quality of available evidence. The panel also agreed that this recommendation is applicable to the Australian context with the majority of Australian patients likely to be in favour of the intervention. The panel agreed that there were no policy or legislative constraints on implementation of this recommendation in Australia, and that there is adequate expertise and equipment in health care settings where the majority of patients typically access DFU care to support implementation of this recommendation.

#### Implementation considerations

##### General considerations

The panel agreed with the IWGDF that all patients with PAD and DFU should be supported to stop smoking, maintain current guideline recommendations for glycaemic and blood pressure control and to take statin and antiplatelet therapy [60]. The panel agreed with the IWGDF that there is no clear evidence in favour of one antiplatelet agent over another, although the panel also agreed that their use individually and in combination is likely to reduce major lower limb events and contribute to a reduction in 5 year mortality [62, 63].

##### Geographically remote people

Relative geographical isolation may reduce access to available support and health education and promotion services required for successful risk factor modification. Referral to appropriate remote support through telehealth and online services should be a priority for patients in these areas.

##### Aboriginal and Torres Strait Islander people

This recommendation is applicable to Aboriginal and Torres Strait Islander people. The panel noted the high prevalence of risk factors for PAD and cardiovascular disease including smoking and hypertension in this population. This highlights the need for establishment of appropriate care referral pathways and care provision to be co-ordinated through Aboriginal Health and Medical Services and for care provision to be supported by an Aboriginal Health Worker to optimise patient outcomes. Further considerations are consistent with those provided for this subgroup in recommendation 11.

For detailed implementation, monitoring and research considerations see eTable C17 in Supplementary Material.

## Discussion

This new Australian guideline for diagnosis and management of PAD in patients with DFU has been developed through a process of reviewing and adopting, adapting or excluding recent international guidelines to meet the needs of the Australian context. This new PAD guideline is one of six new guidelines that together make up the new 2021 Australian evidence-based guidelines for diabetes-related foot disease [64–68] and replace the previous 2011 Australian guidelines [23]. This new guideline includes substantial new evidence relating to diagnosis, prognosis and management in the patient with PAD and DFU. This includes incorporation of new evidence demonstrating the clinical challenge of diagnosing PAD in diabetes cohorts, particularly in relation to the limited capacity of clinical examination (including pulse palpation) and various bedside testing methods to rule out the presence of disease with no single or combination of tests yet to be found to be superior (recommendations 1 to 4, 6 to 8). In addition, the new guideline incorporates the validated WIfI classification system to estimate risk of amputation and potential benefit of revascularisation based on the ulcer characteristics, severity of ischaemia measured via non-invasive bedside testing, and infection severity (recommendation 5). Furthermore, the new guideline provides recommendations regarding revascularisation techniques with limited available evidence and expert opinion favouring direct revascularisation over indirect techniques (recommendation 11). Lastly, the new guideline considers the recommendations in relation to specific subpopulations relevant to the Australian context including those in geographically remote circumstances, and for Aboriginal and Torres Strait Islander people.

While the process of revision and adaptation of existing international guidelines is cost efficient and allows for timely updates, it should also be acknowledged that the adaptation process reduces the capacity firstly to assess new evidence released since publication of the original guideline, and secondly to evaluate available evidence relevant to the Australian context.

### Recommendations and justifications summary

Of the 17 recommendations from the IWGDF Guidelines on diagnosis, prognosis and management of PAD in patients with diabetes and foot ulcers, 16 were adopted for this Australian guideline and one recommendation was adapted. For each of the 16 adopted recommendations the panel agreed with both the strength of the recommendation and the quality of available evidence that was determined by the IWGDF.

Recommendation 13 of the IWGDF Guidelines on diagnosis, prognosis and management of PAD in patients with diabetes and DFU was the only recommendation considered necessary to adapt to the Australian context by the panel. The panel agreed there should be onsite access to appropriate clinical examination and bedside vascular assessment for PAD in any secondary or tertiary centre routinely treating patients with DFU. However, the recommendation in its original form required centres treating DFU to have onsite expertise in diagnosis and treatment of PAD including revascularisation. This was considered by the panel to be unfeasible in Australia. This is due to the geographical expanse of Australia and the smaller populations living in more regional and remote areas challenging the capacity for specialised services and facilities required for advanced diagnosis and surgical interventions for PAD to be available onsite. This recommendation was therefore adapted to include an alternative care model using established referral pathways to ensure rapid access to such facilities and expertise for centres treating DFU in regional and rural Australia.

### Implementation considerations summary

General considerations for implementation related to the limited ability for clinical examination and bedside vascular assessments to rule out PAD in people with diabetes with and without DFU. This highlights the need to undertake further vascular investigation in any patient with DFU where there is evidence of delayed healing (non-healing within 4–6 weeks with optimal care). Further main considerations related to contraindications for specific forms of vascular imaging, for example due to contrast agent allergy or risk of nephrotoxicity, and determination of patient suitability for revascularisation. These factors include poor likelihood of achieving DFU healing or inevitable major amputation, significant risk posed by anaesthesia and the surgical procedure due to the presence of comorbidities including renal disease and infection, the presence of large areas of tissue loss preventing restoration of a functional foot, incapacity for subsequent mobilisation, as well as poor functional status and short life expectancy independent of the presenting DFD.

For geographically remote people, implementation considerations were predominantly in relation to care access. The panel considered it is likely that people in remote areas may experience delayed access to conservative care, particularly in relation to receiving ongoing conservative wound management. The need for early diagnosis of PAD in all patients with diabetes and DFU is paramount. Therefore the panel agreed that further investigation should be undertaken where there is delayed healing without signs of other factors known to impact the healing response such as infection even when there is less regular conservative wound care due to geographical isolation.

Similarly, access to advanced diagnostic services (i.e. vascular imaging) and surgical revascularisation for geographically remote people is likely to be an ongoing challenge to ensuring best outcomes in this population. As discussed previously, rapid referral pathways are required to treatment centres offering revascularisation procedures. Care models inclusive of access to appropriate follow-up assessment and care need to be established in conjunction with involved health care providers. Additional options to support health practitioners in remote areas with appropriate expertise via telehealth and other forms of remote monitoring should be also be considered. Future funding priorities should support strengthening of diabetes-related health care networks across rural and regional Australia to improve provision of, and access to, cohesive care models for PAD and DFU that incorporate appropriate diagnostic, surgical, and conservative management services.

Ensuring adequate access to relevant health services was also considered to be a priority for Aboriginal and Torres Strait Islander people in rural and remote areas where the same restrictions created by geographic isolation occur. In addition, access to culturally safe care is inconsistent across Australia. Distrust of Western health service delivery models has been documented in Aboriginal and Torres Strait Islander people. This is linked to historical and current issues of dispossession and socioeconomic inequality, concern over being removed from family and Community for treatment, along with lack of improvement in Aboriginal and Torres Strait Islander health outcomes through a Western model of health care delivery [69]. Recent research has demonstrated high uptake of preventative DFU care when delivered in a culturally safe manner through a co-designed footcare service developed with an Aboriginal and Torres Strait Islander Community [70]. This emphasises the need for establishment of appropriate care models and related referral pathways that incorporate Community-linked Aboriginal Health and Medical Services and Aboriginal Health Workers.

### Monitoring considerations summary

Monitoring and evaluation is an essential component of establishing best-practice clinical management of DFU. The panel encourages organisations to include in their formal monitoring systems options to be able to collect, monitor and analyse revascularisation and DFU healing outcomes in accordance with national based High Risk Foot Service database monitoring systems and datasets [54–56]. In addition, within services, collection of existing monitoring data from their local hospital discharge datasets also using Australian Classification of Health Interventions codes for specific surgical interventions for PAD is encouraged.

### Future research considerations summary

Fourteen of the 17 recommendations adopted and adapted as part of this revised guideline are supported by low quality of available evidence. The panel agreed with the IWGDF on a number of key priorities for further research. In brief, in relation to bedside testing, there is a need for high quality studies investigating the diagnostic accuracy of bedside testing techniques for diagnosing PAD in people with DFU. Further, the panel agreed with the IWGDF that well-designed prospective research, use of standardised datasets, and the development of international registries are required to more thoroughly assess the predictive capacity of individual and combinations of bedside testing techniques for ischaemic DFU healing outcomes and amputation risk. The panel also agreed with the IWGDF that there is a need for further investigation of novel methods of assessment of perfusion (both micro- and macrovascular) to inform decisions to revascularise. The most effective methods or combination of methods for obtaining imaging of tibial and pedal arteries is of particular importance. This is due to the predilection for a more distally distributed disease pattern in diabetes cohorts, and the increasing use of the angiosome-directed approach to revascularisation where there is direct revascularisation to the feeding artery at the anatomical site of the DFU.

Regarding revascularisation, there is a strong need for high quality evidence to determine optimal time frames for intervention with revascularisation to achieve the best healing outcomes for ischaemic and neuro-ischaemic DFU. The panel agreed with the IWGDF on the need for high level evidence comparing outcomes for angiosome-directed revascularisation compared to indirect revascularisation using both open and endovascular techniques, via randomised controlled trials using pre-defined and standardised outcomes for wound healing and limb salvage [20]. In addition, the proportion of patients with DFU and co-morbidities, including cardiovascular and renal disease, that require revascularisation is rising. As many of these patients are unsuitable for revascularisation, or, are at higher risk of perioperative mortality, the panel agreed with the IWGDF that further research is also required to establish the effectiveness of venous arterialisation for DFU healing and reducing rates of amputation in patients unsuitable for standard revascularisation.

Finally, specific to the Australian context, the panel agreed there is an urgent need for further prospective research investigating DFU healing and limb salvage outcomes in rural and remote areas where accessibility of health care continues to contribute to rural health disparities. For Aboriginal and Torres Strait Islander Communities, achieving better health outcomes for those with PAD and DFU requires a multifaceted approach led by First Nations people to establish a more comprehensive understanding of the extent of PAD in those with DFU, and to undertake prospective evaluation of both models of care delivery, and intervention outcomes for this population.

## Conclusion

This new Australian guideline, adapted from the IWGDF 2019 Guideline on the diagnosis, prognosis and management of PAD in patients with foot ulcers in diabetes, provides a current and comprehensive synthesis of the literature. Modified to suit the Australian context, and in consideration of specific patient subgroups including those in geographically remote areas and Aboriginal and Torres Strait Islander people, the 17 recommendations and the accompanying clinical pathways provide a guide to assist practitioners in secondary and tertiary settings with the implementation of best practice management for patients with diabetes, PAD and DFU. This guideline also highlights the limited available evidence informing strategies for the diagnosis and management of PAD in patients with DFU and the need for future high quality studies of effectiveness of diagnostic accuracy and vascular interventions to reduce amputation rates in non-Indigenous and Aboriginal and Torres Strait Islander people.

## Supplementary Information

**Additional file 1.**

## Data Availability

Data sharing is not applicable to this article as no datasets were generated or analysed during the current study.

## Abbreviations

ABI: Ankle-brachial index
CDUS: Colour duplex ultrasound
CTA: Computed tomography angiography
DFD: Diabetes-related foot disease
DFU: Diabetes-related foot ulcer
DSA: Digital subtraction angiography
EtD: Evidence to Decision
ESRD: End-stage renal disease
GRADE: Grading of Recommendations Assessment, Development and Evaluation
IWGDF: International Working Group on the Diabetic Foot
MRA: Magnetic resonance angiography
NHMRC: National Health and Medical Research Council
TcPO_2_: Transcutaneous oxygen pressure
TBI: Toe-brachial index
WIfI: Wound, Ischaemia, and foot Infection
