# Supplementary material for: Australian guideline on diagnosis and management of peripheral artery disease: part of the 2021 Australian evidence-based guidelines for diabetes-related foot disease
Source: J Foot Ankle Res. 2022 Jul 5;15:51. doi: 10.1186/s13047-022-00550-7 (PMC9254685; doi:10.1186/s13047-022-00550-7)
Supplement: Supplementary file 1 — Additional file 1. [file 13047_2022_550_MOESM1_ESM.docx]

**Online-Only Supplementary Material for:**

**Australian guideline on diagnosis and management of peripheral artery disease: Part of the 2021 Australian evidence-based guidelines for diabetes-related foot disease**

Chuter V, Quigley F, Tosenovsky P, Ritter JC, Charles J, Cheney J, Fitridge R, on behalf of the Australian Diabetes-related Foot Disease Guidelines & Pathways Project

**Table of contents Page**

**APPENDIX A: Tables of detailed justifications for recommendations** 2

1. **DIAGNOSIS 2**

**eTable A1:** Detailed justifications for Recommendation 1 2

**eTable A2:** Detailed justifications for Recommendation 2 4

**eTable A3:** Detailed justifications for Recommendation 3 6

1. **PROGNOSIS 8**

**eTable B4:** Detailed justifications for Recommendation 4 8

**eTable B5:** Detailed justifications for Recommendation 5 10

**eTable B6:** Detailed justifications for Recommendation 6 11

**eTable B7:** Detailed justifications for Recommendation 7 13

**eTable B8:** Detailed justifications for Recommendation 8 15

**eTable B9:** Detailed justifications for Recommendation 9 17

**eTable B10:** Detailed considerations for Recommendation 10 19

1. **TREATMENT 21**

**eTable C11:** Detailed considerations for Recommendation 11 22

**eTable C12:** Detailed considerations for Recommendation 12 24

**eTable C14:** Detailed considerations for Recommendation 14 25

**eTable C15:** Detailed considerations for Recommendation 15 26

**eTable C16:** Detailed considerations for Recommendation 16 27

**eTable C17:** Detailed considerations for Recommendation 17 28

**REFERENCES 30**

**APPENDIX A: Tables of detailed justifications for recommendations**

## DIAGNOSIS

#### Recommendation 1

Examine the feet of all patients with diabetes annually for the presence of peripheral artery disease even in the absence of foot ulceration. At a minimum, this should include taking a relevant history and palpating foot pulses. (Strength of the recommendation: strong; quality of the evidence: low)

**eTable A1:** Implementation, monitoring and research considerations for Recommendation 1

| Topic | Considerations |
| --- | --- |
| General implementation | There are currently limited data investigating the diagnostic accuracy of signs/symptoms or pulse palpation for PAD. One study in 68 people with diabetes without DFU reported the test to have low specificity (i.e. pulses were present in those with significant PAD). In addition, the ABI had high rates of false positive results where pulses were absent is limbs without disease when compared to colour duplex ultrasound (CDUS) which was used as the reference standard (1). In people with diabetes, PAD is frequently asymptomatic or has atypical symptoms (2, 3). While this recommendation is applicable to all people with diabetes without foot ulcer, where there are clinical signs and symptoms of PAD, more frequent screening may be necessary. The high incidence of cardiovascular disease co-existing with PAD necessitates additional cardiovascular risk management in this population to reduce risk of myocardial infarction and stroke (4). |
| Geographically remote people | Given that a range of health professionals have the expertise to conduct a clinical examination for PAD including history taking and pulse palpation, the panel considered that such service should be available in more geographically remote areas |
| Aboriginal & Torres Strait Islander people | This recommendation is applicable to Aboriginal and Torres Strait Islander people. More frequent screening may be appropriate in this population. In addition to a five- to ten-fold increased risk of diabetes in Aboriginal and Torres Strait Islander people, there is a higher incidence of other risk factors for PAD including hypertension and smoking (5, 6). Risk of PAD in Aboriginal and Torres Strait Islander people has been estimated to be three-fold that of non-Indigenous Australians, with evidence of a five-fold increase in prevalence in those undergoing major amputation (7, 8). Basic PAD screening can be provided by a range of health professionals including appropriately trained Aboriginal Health Workers. This may assist in timely screening being provided to Aboriginal and Torres Strait Islander communities in more geographically remote areas.The panel agreed on the high importance of involving an Aboriginal Health Worker in care delivery. The panel also agreed on the importance of explaining the need for, and nature of, the assessment and discussing the results with the patient and their family using a professional interpreter when required. |
| Monitoring & evaluation | The panel felt there were no specific monitoring implications for this recommendation, however, advise to consider the general monitoring implications for the chapter when implementing this recommendation. |
| Future research priorities | As diabetes-related peripheral sensorimotor neuropathy may mask pain symptoms, and presence of autonomic neuropathy can result in a warm foot, this means that the widely recognised signs and symptoms of PAD may not be present (2, 3, 9). Therefore further research is required to determine the diagnostic accuracy of careful history taking and the use of pulse palpation to identify those likely to have PAD, and to better clarify the nature and extent of atypical signs and symptoms and their relationship with co-existent diabetes complications. In addition, due to the cost-effective nature of this strategy, and the capacity to use this widely at a community level across diverse geographical regions and via a range of health practitioners, prospective investigation of the effectiveness of this screening for preventing development of DFD and reducing rates of amputation is required. |

#### Recommendation 2

Clinically examine (by relevant history and palpation of foot pulses) all patients with diabetes and foot ulceration for the presence of peripheral artery disease. (Strong; low)

**eTable A2:** Implementation, monitoring and research considerations for Recommendation 2

| Topic | Considerations |
| --- | --- |
| General implementation | As per recommendation 1, presence of co-existent microvascular disease in the form of peripheral neuropathy can result in loss of peripheral pain perception, while autonomic neuropathy is implicated in the development of arteriovenous shunting which results in a warm foot (9-11). These factors may mask pain symptoms and prevent temperature loss in the periphery that are typical of PAD. Careful history taking and palpation of pulses is an important initial step in the examination of a patient with a DFU. In addition, a relatively recent study investigated specific aspects of clinical examination including clinical signs such as hair growth, skin temperature and colour and palpation of pulses for diagnosis of PAD confirmed by CDUS. Of note the sensitivity and specificity of pulse palpation for diagnosis PAD was found to be low and insufficient to rule out the disease (12). Similarly none of the clinical signs of PAD were found to be of adequate diagnostic accuracy for PAD. Therefore results of clinical examination of the foot (from patient history and pulse palpation) should be considered in combination with additional objective testing. |
| Geographically remote people | As per recommendation 1, given that a range of health professionals have the expertise to conduct a clinical examination for PAD including history taking and pulse palpation, the panel considered that such service should be available in more geographically remote areas |
| Aboriginal & Torres Strait Islander people | This recommendation is applicable to Aboriginal and Torres Strait Islander people. More frequent screening may be appropriate in this population as per recommendation 1. The panel noted that due to the heightened risk of poor outcomes for DFU in Aboriginal and Torres Strait Islander people and the increased likelihood of PAD in this population (13), clinical examination that does not identify risk of PAD should be treated with an abundance of caution. It is particularly important in this population that further bedside testing is conducted as an adjunct to basic clinical examination. Basic PAD screening can be provided by a range of health professionals including appropriately trained Aboriginal Health Workers. This may assist in timely screening being provided to Aboriginal and Torres Strait Islander communities in more geographically remote areas.The panel agreed on the high importance of involving an Aboriginal Health Worker in care delivery. The panel also agreed on the importance of explaining the need for, and nature of, the assessment and discussing the results with the patient and their family using a professional interpreter when required. |
| Monitoring & evaluation | The panel felt there were no specific monitoring implications for this recommendation, however, advise to consider the general monitoring implications for the chapter when implementing this recommendation. |
| Future research priorities | As per recommendation 1, there are little available data investigating the accuracy of signs and symptoms or clinical examination (e.g. history taking, pulse palpation) for identifying the presence of PAD in people with diabetes with DFU. Significantly, as 50% of people with DFU have been shown to have co-existing PAD, the likelihood of such testing being able to confidently rule out PAD is low, and therefore should be used in conjunction with bedside testing (12, 14, 15). However, further research is required to determine the effect on diagnostic accuracy of clinical examination and bedside PAD testing when used in specific combinations. The use of clinical examination (history taking and pulse palpation) in conjunction with other forms of bedside testing such as the ABI may improve the capability of such testing to rule PAD diagnosis out. |

#### Recommendation 3

As clinical examination does not reliably exclude PAD in most persons with diabetes and a foot ulcer, evaluate pedal Doppler arterial waveforms in combination with ankle systolic pressure and systolic ankle brachial index (ABI) or toe systolic pressure and toe brachial index (TBI) measurement. No single modality has been shown to be optimal, and there is no definite threshold value above which PAD can reliably be excluded. However, PAD is a less likely diagnosis in the presence of ABI, 0.9-1.3; TBI, ≥ 0.75; and triphasic pedal Doppler waveforms. (Strong; low)

**eTable A3:** Implementation, monitoring and research considerations for Recommendation 3

| Topic | Considerations |
| --- | --- |
| General implementation | A range of health professionals are able to undertake bedside diagnostic vascular testing of the lower limb. Provision of appropriate equipment and training to health professionals caring for people with DFU is necessary to ensure adequate testing can be conducted. Of note there is not enough evidence to determine if there is any single, or combination of bedside tests, which has greater diagnostic accuracy for PAD. Therefore, choice of test (or tests) should be made based on available equipment and expertise at any given location. The majority of available studies are observational, have moderate to high risk of bias and use a variety of definitions of disease in the reference test and varying diagnostic thresholds for the index test (16). There have been few diagnostic accuracy studies performed in people with diabetes with an active foot ulcer, with most related to the ABI (16). The majority of these studies use CDUS as reference standard. This form of testing may be less reliable for diagnosing PAD severity in tibial vessels which are commonly affected in diabetes-related PAD and in vessels affected by significant calcification which is an additional complication of diabetes (16). It is therefore essential that health care professionals recognise that all the available testing modalities have specific limitations to the capacity of each of these tests to accurately identify the presence of significant PAD. Furthermore, while not within in the scope of this guideline, application of testing should be consistent with current recommendations using standardised protocols (17-19). These include allowing adequate pretest rest time, ensuring appropriate patient positioning, equipment and control of environmental and extrinsic factors known to affect the measurements e.g. cold temperatures, exercise, smoking or caffeine consumption immediately prior to testing (19-22) Presence of diabetes-related medial artery calcification (MAC), which is estimated to affect 30-40% of people with diabetes, is known to affect the accuracy of some bedside tests including the ABI and, to a lesser extent the TBI (15, 23). Calcification of arteries in the leg and toe may prevent compression of the vessel and create an artificially high systolic pressure measurement. In the case of the ABI an internationally recommended established upper threshold of 1.3 is used, with results in excess of this considered indicative of MAC and a prompt for additional alternate testing (24). Although there is an established upper threshold for the ABI the potential co-existence of both MAC and PAD and the effect of this on ABI results should also be recognised. For example, gangrene and digital and below knee amputations have also been demonstrated to occur in patients with ABI values in excess of 1.40 indicating that in some situations both disease processes co-exist and are severe (25). In addition, in 187 participants with diabetes who were assessed by ABI significant associations were found between the presence of MAC and greater severity of atherosclerosis and a lower ABI (26). The latter is contrary to the expected elevated pressures in this cohort, and supports the suggestion that the ABI can be elevated into normal range in the presence of both MAC and PAD and is consistent with findings of limited diagnostic accuracy of the ABI for PAD in people with diabetes (27). These findings support the need for additional forms of testing.  For the TBI there is currently no established upper limit for the test making determining presence of digital arterial calcification more difficult to determine and supporting the use of several bedside tests in parallel. |
| Geographically remote people | There may be restricted access to appropriate expertise and equipment in geographically remote areas. However the panel felt that where there are existing health services providing DFU treatment and management, required bedside testing should be available with choice of test (or tests) directed by availability of specific equipment and expertise. |
| Aboriginal & Torres Strait Islander people | This recommendation is applicable to Aboriginal and Torres Strait Islander people. The panel agreed on the high importance of involving an Aboriginal Health Worker in care delivery. The panel also agreed on the importance of explaining the need for, and nature of, the assessment and discussing the results with the patient and their family using a professional interpreter when required. |
| Monitoring & evaluation | The panel encourages organisations to include in their formal monitoring systems options to be able to collect, monitor and analyse bedside diagnostic vascular testing results and DFU healing outcomes in accordance with national based High Risk Foot Service database monitoring systems and datasets (28-30) |
| Future research priorities | There is a dearth of available data investigating the diagnostic accuracy of objective testing measures such as the ABI, TBI, systolic ankle and toe pressures and TcPO_2_ in people with diabetes. Of the available evidence the ABI has been most extensively investigated. A recent systematic review found the ABI to have limited diagnostic accuracy for PAD in people with diabetes with the testing shown to have a low probability of being able to rule diagnosis in or out (27). There are limited data available for the diagnostic accuracy of alternate forms of testing including systolic ankle and toe pressures, the TBI, TcPO_2_ and pulse oximetry. Further high quality diagnostic accuracy studies are required that adequately control for bias, (assessor blinding, partial verification bias and spectrum bias), and that assess the optimal combination of bedside tests for diagnosing PAD in people with diabetes. There are some data indicating combinations of tests in patients with DFD and chronic limb-threatening ischaemia improves diagnostic accuracy of bedside testing (31, 32). Future studies should use standardised methods of measurement performance, consistent methods for diagnosis of PAD with the reference standard, and consistent diagnostic thresholds for the index test. Similarly, the accuracy of such testing methods in subpopulations of people with diabetes including those with peripheral neuropathy, and with and without active DFU, need to be undertaken.  In addition, the panel agreed with the IWGDF that moves away from using fixed thresholds for diagnosing ischaemia with bedside testing methods in favour of classification systems of outcomes related to clinical staging represents an additional key area for further research focus. Beyond diagnosing PAD, the role of these measurements in determining the presence and severity of ischaemia in combination with other relevant contributing factors including the presence of neuropathy and infection for the development and outcomes of DFU also requires more extensive investigation. |

#### Recommendation 4

As clinical examination does not reliably exclude PAD in most persons with diabetes and a foot ulcer, evaluate pedal Doppler arterial waveforms in combination with ankle systolic pressure and systolic ankle brachial index (ABI) or toe systolic pressure and toe brachial index (TBI) measurement. No single modality has been shown to be optimal, and there is no definite threshold value above which PAD can reliably be excluded. However, PAD is a less likely diagnosis in the presence of ABI, 0.9-1.3; TBI, ≥ 0.75; and triphasic pedal Doppler waveforms. (Strong; low) Perform at least one of the following bedside tests in a patient with a diabetes-related foot ulcer and PAD, any of which increases the pretest probability of healing by at least 25%: a skin perfusion pressure of ≥40 mmHg, a toe pressure of≥ 30 mmHg, or a transcutaneous oxygen pressure (TcPO2) of ≥25 mmHg. (Strong; moderate)

**eTable B4:** Implementation, monitoring and research considerations for Recommendation 4

| Topic | Considerations |
| --- | --- |
| General implementation | In a small number of studies (although outcomes are variable) there is evidence of skin perfusion pressure of ≥40 mmHg, toe pressure ≥30 mmHg, orTcPO_2_ ≥25 mmHg have individually been shown to increase the probability of foot ulcer healing by more than 25% (33). The panel considered that these findings suggest the above thresholds are useful in determining patient suitability for initial implementation of conservative therapy prior to considering revascularisation. This is on the provision that the results of assessment of peripheral perfusion are considered in the context of the presence or absence of other factors, for example infection, which may further impede healing. In addition, in circumstances where there are pressures above these bedside testing thresholds, due to limitations in all the diagnostic testing methods that are recommended (TcPO_2_, skin perfusion pressure and toe pressures), and the lack of consistency in their accuracy for predicting healing in the literature, PAD should not be excluded as a contributor to poor wound healing when there is a lack of response to optimal care (24). Similarly where there are other factors indicating poor healing prognosis, including presence of extensive infection or large wound surface area, urgent imaging and potential revascularisation should still be considered (34).  The bedside testing recommended requires specific equipment and expertise, and results should be considered in conjunction with clinical examination findings. Skin perfusion pressure is typically measured via laser Doppler which has been shown to have high accuracy compare to gold standard radioisotopic clearance historically used to measure skin perfusion pressure (35). Although laser Dopplers are portable and are relatively simple to use, the equipment is of significant cost, and the reliability of measures of microvascular function is variable (36). TcPO_2_ also requires specialised equipment of significant cost and time investment to conduct the measurements. In the alternative, toe pressures can be reliably conducted using manual or automated equipment with variable, but far lower, equipment costs and shorter times required for measurements to be conducted (37, 38). Therefore the panel considered that centres treating people with DFU would have access to equipment to perform one or more of these tests on the majority of patients.  In situations where there is a hallux (or first digit) ulcer, or there has been previous hallux amputation, using the second toe for pressure measurement has been shown to be a valid alternate measure to hallux pressures although consideration needs to be given to cuff size (39). Where there is amputation /ulcer involving both first and second digits, toe pressures are not possible. In such circumstances TcPO_2_ or skin perfusion pressure are required. If these measurements are not feasible due to lack of equipment or expertise, an ankle pressure or the ABI can be used to confirm if there is increased likelihood of amputation. Neither ankle pressures nor the ABI have been demonstrated to be predictive of ulcer healing, however, an ankle pressure of <50 mmHg and an ABI of <0.5 have both been shown to be associated with increased risk of amputation (34, 40). |
| Geographically remote people | Lack of specialised equipment, particularly for measuring skin perfusion pressure and TcPO_2_, may limit choice of testing being conducted in remote areas. However, as health care centres treating people with DFU in remote areas should routinely be performing bedside testing for PAD in patients, toe pressures are therefore a suitable alternative measurement. |
| Aboriginal & Torres Strait Islander people | This recommendation is applicable to Aboriginal and Torres Strait Islander people. However the need to consider the results of the vascular testing performed within the context of other risk factors for non-healing in the population is particularly important. To the panel’s knowledge there are currently no data investigating the capacity for skin perfusion pressure, TcPO_2_ and toe pressure to predict likelihood of DFU healing in Aboriginal and Torres Strait Islander people. Similarly there are little data investigating specific factors contributing to non-healing and amputation outcomes in this population. The available evidence suggests Aboriginal and Torres Strait Islander people have a four-fold increase in risk of diabetes-related peripheral neuropathy, a five- and six-fold increase in risk of foot ulcer and amputation respectively, and, that they are more likely to have PAD but less likely to have had it diagnosed (13). For Aboriginal and Torres Strait Islander people, rates of DFD are higher in rural and remote communities with this population comprising up to 91% of those undergoing amputation in these areas (41-43). The heightened risks of both concurrent intrinsic (e.g. peripheral neuropathy) and extrinsic (e.g. health care access) risk factors for non –healing for people in rural and regional areas, need to be considered when assessing probability of healing and in the implementation of a management plan. |
| Monitoring & evaluation | Panel monitoring and evaluation advice is as per recommendation 4 |
| Future research priorities | Further research is required to more conclusively determine the predictive capacity of bedside testing for wound healing in patients with a DFU. Outcomes of available research are highly variable and there are a lack of data available for specific populations including Aboriginal and Torres Strait Islander people who experience disproportionately high rates of non–healing and amputation. In addition, the role of other methods of measurements of macro- and microvascular function in the lower extremity to inform decisions on revascularisation need investigation. For example, measures of microvascular reactivity including post-occlusive reactive hyperaemia measured via laser Doppler in the foot has been proposed to be associated with the vasodilatory capacity of the microvasculature and therefore the healing response (44). In addition, perfusion angiography provides quantitative data relating to regional tissue perfusion and can be related to the DFU site, and may also be predictive of DFU healing capacity (45). |

#### Recommendation 5

Use the Wound, Ischaemia, and foot Infection (WIfI) classification system as a means to stratify amputation risk and revascularisation benefit in a patient with a diabetes-related foot ulcer and PAD. (Strong; moderate)

**eTable B5:** Implementation, monitoring and research considerations for Recommendation 5

| Topic | Considerations |
| --- | --- |
| General implementation | Given the availability of the WIfI tool, and its use of non-invasive bedside testing to determine level of ischaemia, and clinical grading of infection and the wound, the panel agreed there would be no specific limitations to implementation. However, the capacity of the WIfI tool to determine amputation risk and benefit of revascularisation may be affected in patients with reduced peripheral perfusion from other causes. For example both peripheral oedema and presence of infection may cause a reduction in tissue oxygenation that is typically seen with ischaemia due to PAD. In such circumstances these conditions will require appropriate intervention (24). |
| Geographically remote people | The panel agreed that this recommendation is suitable for implementation in geographically remote areas and that the nature of the classification system, including use of bedside testing and clinical grading of wound and infection severity, makes its suitable for use in geographically isolated areas. The panel noted that the likelihood of DFU healing following classification is affected by the quality of care and capacity for that care to address problems identified by the tool. Therefore clinical decision making based on the WIfI classification system in geographically remote areas would also need to be informed by access to ongoing wound care services and individual socioeconomic circumstances of the patient. |
| Aboriginal & Torres Strait Islander people | The panel agreed that this recommendation is generally applicable to Aboriginal and Torres Strait Islander people. As the WIfI tool has not been validated in this specific population, as per recommendation 4, the disproportionately high risk of amputation in Aboriginal and Torres Strait Islander people particularly in rural and remote areas, and extrinsic and cultural barriers to care access (for example the need to stay on country, family and community circumstances and roles, and a potential preference for community delivered care) need to be considered in addition to the WIfI classification system to better determine risk of amputation and benefits of revascularisation. |
| Monitoring & evaluation | The panel encourages organisations to include in their formal monitoring systems options to be able to collect, monitor and analyse WIfI classification and revascularisation and DFU healing outcomes in accordance with national based High Risk Foot Service database monitoring systems and datasets (28-30). |
| Future research priorities | Further validation of the WIfI tool in the Australian context and in subpopulations particularly at risk of DFU and amputation, for example Aboriginal and Torres Strait Islander people, is required to increase the strength of this recommendation and better inform specific subgroup considerations. |

#### Recommendation 6

Always consider urgent vascular imaging, and revascularisation, in a patient with a diabetes-related foot ulcer and an ankle pressure of <50 mmHg, ABI of <0.5, a toe pressure of <30 mmHg, or a TcPO2 of <25 mmHg. (Strong; low)

**eTable B6:** Implementation, monitoring and research considerations for Recommendation 6

| Topic | Considerations |
| --- | --- |
| General implementation | As noted in recommendation 5, although ankle pressures and the ABI have not been demonstrated to be predictive of ulcer healing, an ankle pressure of <50 mmHg and an ABI of <0.5 have been shown to be associated with increased risk of amputation supporting the using of urgent vascular imaging and revascularisation in such circumstances (33). Similarly a toe pressure of <30 mmHg, or a TcPO_2_ of <25 mmHg have both been linked to poor healing outcomes and increased risk of amputation, supporting the need for rapid vascular imaging and potential revascularisation in such patients (33).  The limited data investigating the natural progression of ischaemic DFU support a healing rate of approximately 50% (46, 47). Therefore in some patients with an ankle pressure of<50 mmHg, ABI of <0.5, a toe pressure of <30 mmHg, or a TcPO_2_ of <25 mmHg where there is unacceptable risk presented by a revascularisation process e.g. from anaesthesia or comorbidities, conservative treatment should be considered. |
| Geographically remote people | This recommendation is applicable to geographically remote locations, however, in these situations timely referral for imaging and revascularisation requires well established rapid referral pathways which should be developed in consideration of the specific circumstances of different geographically remote regions. |
| Aboriginal & Torres Strait Islander people | This recommendation is applicable to Aboriginal and Torres Strait Islander people. The reader is referred to considerations in recommendation 1 and 2. The panel also agreed on the importance of explaining the need for, and nature of, any further vascular intervention or surgical intervention including the expected timeframes for, and location of, related hospitalisation and longer term post-operative care with the patient and their family using a professional interpreter when required. Furthermore due to the disproportionately high risk of amputation in Aboriginal and Torres Strait Islander people particularly in rural and remote areas, extrinsic and cultural barriers to care access need to be considered in the establishment of appropriate rapid referral pathways, and in considering revascularisation procedures. |
| Monitoring & evaluation | The panel encourages organisations to include in their formal monitoring systems options to be able to collect, monitor and analyse bedside vascular testing results and DFU healing outcomes in accordance with national based High Risk Foot Service database monitoring systems and datasets (28-30). |
| Future research priorities | Most available data assessing risk of non-healing and amputation based on bedside vascular testing results fail to adequately account for confounders for wound healing. The panel agrees with the IWGDF that further, well-designed prospective research, use of standardised data sets, and the development of international registries are required to more thoroughly assess the predictive capacity of individual and combinations of bedside testing techniques for ischaemic DFU healing outcomes and risk of amputation. Conducting such research in subgroups at higher risk of amputation particularly in the Australian context including Aboriginal and Torres Strait Islander people and those in remote areas should also be undertaken. |

#### Recommendation 7

Always consider vascular imaging in patients with a diabetes-related foot ulcer, irrespective of the results of bedside tests, when the ulcer is not healing within 4 to 6 weeks despite good standard of care. (Strong; low)

**eTable B7:** Implementation, monitoring and research considerations for Recommendation 7

| Topic | Considerations |
| --- | --- |
| General implementation | As discussed in recommendation 3, the paucity of available research investigating diagnostic accuracy of bedside testing for PAD in patients with DFU highlights the limited capacity for this testing to rule out presence of the disease. Undiagnosed ischaemia is therefore a potential contributing factor to delayed healing in situations where appropriate conservative care is being provided. Current available evidence suggests the timeframe for implementing additional vascular imaging and undertaking revascularisation where appropriate influences healing outcomes (48). There is recent retrospective evidence demonstrating that a delay in revascularisation of more than 2 weeks post presentation of a DFU results in increased risk of limb loss (48). This is supported by observational research showing an increase in likelihood of healing of ischaemic foot ulcers where revascularisation occurs more rapidly and less than 8 weeks after presentation.  In patients where the DFU is being managed conservatively due short life-expectancy independent of the presenting DFD, the panel agreed that vascular imaging was unlikely to provide any additional benefit to the patient. |
| Geographically remote people | Differing levels of accessibility to conservative DFU care in remote regions may affect ulcer healing outcomes including time to achieve healing. Gold standard multidisciplinary DFU care involves regular debridement and wound dressing, as well as effective offloading and rapid control of the presence of infection. In more geographically remote areas delays or more extended time between appointments as well as hot or dry and dusty environments may reduce adherence to some conservative therapies (for example to offloading devices) which may also slow the healing time. Nevertheless, due to the need to diagnose PAD as soon as possible where delayed healing is occurring, further imaging should be sought. |
| Aboriginal & Torres Strait Islander people | This recommendation is applicable to Aboriginal and Torres Strait Islander people. The reader is referred to considerations in recommendation 1 and 2 and to the considerations for those living in geographically remote areas as described above, and in recommendation 6. In addition, the panel agreed that the need to remove protective offloading devices for community-based cultural activities may adversely affect the healing rate of DFU in this population. However due to the high incidence of PAD in Aboriginal and Torres Strait Islander People, further imaging should be sought where there is practitioner concern over healing response. |
| Monitoring & evaluation | The panel encourages organisations to include in their formal monitoring systems options to be able to collect, monitor and analyse bedside vascular testing results and DFU healing outcomes in accordance with national based High Risk Foot Service database monitoring systems and datasets (28-30). |
| Future research priorities | As per recommendation 3 the panel agrees with the IWGDF that further studies are required to determine the most effective combination of bedside testing to both diagnose PAD and predict healing in DFU. Using serial or parallel testing with several modalities may increase the capacity to exclude the presence of PAD making it less likely to be present when accompanied by normal test results. In addition, the panel agreed with the IWGDF on the pragmatic approach of a four- to six-week healing timeframe to be expected in a DFU where there are no other obvious factors for poor wound healing. However several studies have demonstrated that a shorter time to revascularisation is associated with higher probability of DFU healing and lower likelihood of limb loss (48, 49). Therefore further investigation of the most appropriate wound healing measure, for example percentage of wound size reduction or complete healing, to prompt further vascular imaging is also warranted. |

#### Recommendation 8

Always consider revascularisation in a patient with a diabetes-related foot ulcer and PAD, irrespective of the results of bedside tests, when the ulcer is not healing within 4 to 6 weeks despite optimal management. (Strong; low).

**eTable B8:** Implementation, monitoring and research considerations for Recommendation 8

| Topic | Considerations |
| --- | --- |
| General implementation | There is recent retrospective evidence demonstrating that a delay in revascularisation of more than two weeks post presentation of a DFU results in increased risk of limb loss (48). This is supported by observational research showing an increase in likelihood of healing of ischaemic foot ulcers where revascularisation occurs more rapidly and less than eight weeks after presentation (49). These findings highlight the need to ensure timely referral of patients into vascular assessment and treatment services where there is evidence of delayed healing of DFU in the absence of other variables likely to be contributing to poor wound healing. In patients where the DFU is being managed conservatively due to short life expectancy independent of the presenting DFD, the panel agreed that vascular imaging was unlikely to provide any additional benefit to the patient. |
| Geographically remote people | As per recommendation 7 difficulties for patients regularly accessing optimal conservative care either due to distance or service availability may contribute to delayed healing and in these cases highlights the need for individual patient circumstances and results of vascular imaging to be used to inform decisions relating to revascularisation. . |
| Aboriginal & Torres Strait Islander people | This recommendation is applicable to Aboriginal and Torres Strait Islander people with specific considerations for this recommendation consistent with those outlined in recommendations 1, 2, and 6 in relation to care delivery involving an Aboriginal Health Worker and the need for effective patient and family communication regarding assessment and treatment options. As per recommendation 7, the panel noted Aboriginal and Torres Strait Islander people may experience reduced frequency of access to appropriate care due to cultural barriers and lack of culturally safe care as well as difficulty due to geographical remoteness. This may have an adverse effect on healing rates and as with those living in remote geographical areas such circumstances should be considered in addition to vascular imaging when considering revascularisation |
| Monitoring & evaluation | The panel encourages organisations to include in their formal monitoring systems options to be able to collect, monitor and analyse revascularisation and DFU healing outcomes in accordance with national based High Risk Foot Service database monitoring systems and datasets (28-30). In addition, within services, collection of existing monitoring data from the local hospital discharge datasets using Australian Classification of Health Interventions codes for specific surgical interventions relating to PAD is encouraged. |
| Future research priorities | The panel agreed with the pragmatic timeframe of four to six weeks for improvement in neuro-ischaemic ulcers where no additional factors contributing to poor wound healing are present and optimal care is provided, as recommended by the IWGDF. There is some evidence to indicate that percentage of ulcer reduction over four weeks is sufficient to predict healing of non-complicated neuropathic DFU at 12 weeks (50). However several studies have reported variable time periods to revascularisation of between two and eight weeks as being associated with higher probability of DFU healing and lower likelihood of limb loss (48, 49).  Therefore further prospective investigation of optimal timeframes for intervention with revascularisation to achieve the best healing outcomes is warranted for ischaemic and neuro-ischaemic DFU. |

#### Recommendation 9

Do not assume diabetes-related microangiopathy, when present, is the cause of poor healing in patients with a diabetes-related foot ulcer; therefore, always consider other possibilities for poor healing. (Strong; low)

**eTable B9:** Implementation, monitoring and research considerations for Recommendation 9

| Topic | Considerations |
| --- | --- |
| General implementation | Diabetes-related microangiopathy is associated with a myriad of conditions including those affecting the eyes, kidneys, cardiac muscle, skin and neurological system and is characterised by increased capillary basement membrane thickening. Via a pilot randomised controlled trial additional arteriolar occlusions in the foot have been shown in patients with diabetes with neuroischaemia compared to those with neuropathy alone (51). The apparent additional small vessel disease is suggested to delay wound healing but have no effect on outcomes of revascularisation or risk of amputation. Previously autonomic neuropathy has been proposed to cause dysregulation of the arterio-venous anastomoses, resulting in loss of sympathetic tone and relaxation of the shunts which diverts blood away from the skin and contributes to localised tissue hypoxia (Capillary Steal Syndrome) (52). Evidence of progressive increases in arterio-venous shunt flow in the presence of diabetes alone, diabetes with neuropathy, and diabetes with neuropathy and foot ulceration supports the presence of this mechanism of dysfunction and the role it may play in foot complications (11). However, due to the lack of compelling evidence supporting a role of microangiopathy in poor DFU healing, the panel agreed with the IWGDF that other factors that may impair wound healing and reduce peripheral perfusion including PAD undiagnosed by bedside testing, presence of oedema and infection should be considered first and foremost. |
| Geographically remote people | The panel consider this recommendation to be applicable to people living in geographically remote areas. The panel noted the importance of thorough investigation of both intrinsic (e.g. infection, PAD) and extrinsic (e.g. access to care) factors associated with delayed healing in geographically remote people. |
| Aboriginal & Torres Strait Islander people | The panel considered this recommendation to be suitable for Aboriginal and Torres Strait Islander people, but, as per recommendations 6 and 7, identified the need to consider extrinsic factors that may contribute to delayed, or non-healing in this population. These include adequate access to culturally safe care, suitability of conservative care to cultural needs and similar potential restrictions in access to regular conservative care in geographically remote areas. |
| Monitoring & evaluation | The panel felt there were no specific monitoring implications for this recommendation, however, advise to consider the general monitoring implications for the chapter when implementing this recommendation. |
| Future research priorities | Further research is required to establish the extent of any role of microangiopathy in DFU healing. While peripheral neuropathy is well established as a risk factor for developing DFU the role of cutaneous microangiopathy in DFU development and healing has not been established. Recent research has highlighted histological changes to capillary structure in the foot occurring in association with neuro-ischaemic and neuropathic foot ulcers in an older people with type 2 diabetes (51). These include a reduction in capillary lumen diameter, basement membrane thickening and a reduction in capillary density. Reduced cutaneous vasodilatory capacity has also been found to be associated with the presence of peripheral neuropathy and there is also growing evidence that advanced glycosylated end-products may contribute to capillary wall stiffness, further impairing the vasodilatory response (53). These findings suggest that functional impairment of local vasodilatory capacity of the microvasculature, as well as possible occlusive disease of arterioles may impair DFU healing. Prospective investigations of measures of microvascular function and microvascular reactivity in patients with DFU in tissue local to the wound site with appropriate control of confounding variables known to influence wound healing are required. |

#### Recommendation 10

Use any of the following modalities to obtain anatomical information when considering revascularising a patient's lower extremity: colour duplex ultrasound (CDUS), computed tomographic angiography (CTA), magnetic resonance angiography MRA), or intra-arterial digital subtraction angiography (DSA). Evaluate the entire lower extremity arterial circulation with detailed visualization of below-the-knee and pedal arteries, in an anteroposterior and lateral plane. (Strong; low).

**eTable B10:** Implementation, monitoring and research considerations for Recommendation 10

| Topic | Considerations |
| --- | --- |
| General implementation | CDUS is non-invasive but requires advanced equipment and is reliant on the skills of the sonographer. In addition, in patients where there is diffuse multi-segment disease, extensive calcification or oedema visualisation of arteries is hampered (54). Similarly, CTA which requires intravenous iodated contrast medium, will provide imaging from the renal to pedal arteries. However, this imaging technique is also affected by the presence of calcification, as well as patient specific contraindications to the use of the contrast agent including allergy and risk of contrast induced nephropathy and, is therefore unsuitable in many patients with existing renal disease.  Nephrotoxicity can be reduced with use of contrast enhanced MRA which uses a gadolinium contrast agent. This technique produces imaging from the abdominal aorta to the pedal arteries, however image quality is affected by low spatial resolution and artefacts from previous stent placement. Further contraindications to MRA include severe renal insufficiency (a relative contraindication where a non-gadolinium agent may be required), presence of a pacemaker or some other implants and in claustrophobic patients without sedation. DSA is considered to be gold standard for arterial imaging providing high resolution images and allowing for concurrent endovascular procedures. However this form of imaging is both invasive and uses an iodine-based contrast agent. It is therefore contraindicated where there is allergy, where risk of contrast-induced nephropathy is a relative contraindication and where there is a risk of complications at the arterial access site (24). |
| Geographically remote people | The panel agreed that while a range of imaging services may be available in metropolitan and regional areas, this access is likely to be very limited in geographically remote areas. In such situations the importance of well-established clinical referral pathways to support timely access to services is paramount. |
| Aboriginal & Torres Strait Islander people | The panel considered that this recommendation was appropriate for Aboriginal and Torres Strait Islander people. Consistent with populations in remote geographical areas, the importance of established referral pathways developed in conjunction with community based Aboriginal Health and Medical Services and where the care provision is supported by an Aboriginal Health Worker is integral to optimising patient outcomes. In addition, the reader is referred to considerations for Aboriginal and Torres Strait Islander people for recommendations 1 and 2. |
| Monitoring & evaluation | The panel felt there were no specific monitoring implications for this recommendation, however, advise to consider the general monitoring implications for the chapter when implementing this recommendation. |
| Future research priorities | Due to the specific limitations of the available imaging modalities and the reduced measures of diagnostic accuracy of bedside vascular testing methods, the panel agreed with the IWGDF that there is a need for further investigation of novel methods of assessment of perfusion (both micro and macrovascular) to inform decisions to revascularise. The most effective methods, or combination of methods, for obtaining imaging of tibial and pedal arteries is of particular importance due to the predilection for a more distally distributed disease pattern in people with diabetes and the increasing use of the angiosome-directed approach to revascularisation where there is direct revascularisation to the feeding artery to the anatomical site of the DFU see recommendation 11). |

## TREATMENT

## Recommendation 11

When performing revascularisation in a patient with a diabetes-related foot ulcer, aim to restore direct blood flow to at least one of the foot arteries, preferably the artery that supplies the anatomical region of the ulcer. After the procedure, evaluate its effectiveness with an objective measurement of perfusion. (Strong; low)

**eTable C11:** Implementation, monitoring and research considerations for Recommendation 11

| Topic | Considerations |
| --- | --- |
| General implementation | While the most effective approach to revascularisation remains a point of contention, the panel agreed with the IWGDF that direct revascularisation, where there is restoration of flow to the anatomical area in which the ulcer is located, will theoretically be more effective than an indirect technique. Of the few data available investigating outcomes without revascularisation, several studies have shown amputation rates in populations with diabetes and limb ischaemia are approximately 50% at one year (46, 47). In contrast, revascularisation is associated with a limb salvage rate of up to 85%, with ulcer healing at >60% at one year (4). Using an indirect technique where the best target vessel is used regardless of the ulcer location relies on collateral flow (which is known to be poor in people with diabetes) to increase perfusion to the ulcer site (24).  In addition, the panel agreed with the IWGDF that assessment of blood flow to the ulcer site during the procedure and potentially opening multiple arteries to achieve maximum possible perfusion confirmed via objective testing (e.g. skin perfusion pressure, TcPO_2_) is recommended to ensure a clinically successful outcome. The panel also agreed that following completion of the procedure there is evidence to support progressive increases in perfusion, and therefore with repeat testing between one and three weeks after the procedure should be conducted.  The panel also agreed that in the presence of end-stage renal disease revascularisation needs to be carefully considered due to high rates of complications, a five-year mortality rate of 91% and moderate limb salvages rates (65-70%) for those surviving to one year (4). The panel agreed with the IWGDF that, in the presence of extensive infection, therapy should be implemented to control the infection prior to undertaking a revascularisation procedure and subsequent restoration of perfusion should be undertaken within a few days of stabilisation of the patient (24). |
| Geographically remote people | The panel agreed that this recommendation is applicable to people living in geographically remote areas, the panel noted that, for these patients, rapid referral pathways are required to treatment centres offering direct and indirect revascularisation procedures and that access to appropriate follow-up assessments and care needs to be established as part of the management model in conjunction with involved health care providers. Options to support health practitioners in remote areas with appropriate expertise via telehealth and other forms of remote monitoring should be considered. |
| Aboriginal & Torres Strait Islander people | The panel considered that this recommendation to be applicable Aboriginal and Torres Strait Islander people. The panel agreed on the importance of explaining the need for, and nature of, any surgical intervention including the expected timeframes for, and location of, related hospitalisation and longer-term post-operative care with the patient and their family using a professional interpreter when required. Furthermore, due to the disproportionately high risk of amputation in Aboriginal and Torres Strait Islander people particularly in rural and remote areas, extrinsic and cultural barriers to care access need to be considered in the establishment of appropriate rapid referral pathways and in considering revascularisation procedures. Consistent with populations in remote geographical areas the importance of established referral pathways as well as appropriate culturally safe follow up care in required for Aboriginal and Torre Strait Islander people in all geographical locations. These should be developed in conjunction with community-based Aboriginal Health and Medical Services ideally with the care provision supported by an Aboriginal Health Worker to optimise patient outcomes. |
| Monitoring & evaluation | Panel monitoring and evaluation advice is as per recommendation 8 |
| Future research priorities | Current evidence comparing angiosome directed revascularisation to indirect revascularisation shows the former technique consistently has superior outcomes for wound healing, however outcomes for limb salvage are more variable (20). However, this evidence is based on outcomes from case series and cohort data which have a high risk of bias. The panel agreed with the IWGDF that prospective comparative research needs to be undertaken to investigate the DFU healing outcomes and limb salvage rates associated with angiosome-directed revascularisation compared to indirect revascularisation using both open and endovascular techniques. |

#### Recommendation 12

As evidence is inadequate to establish whether an endovascular, open, or hybrid revascularisation technique is superior, make decisions based on individual factors, such as morphological distribution of PAD, availability of autogenous vein, patient co-morbidities, and local expertise. (Strong; low)

**eTable C12:** Implementation, monitoring and research considerations for Recommendation 12

| Topic | Considerations |
| --- | --- |
| General implementation | The panel agreed with the IWGDF that the nature of diabetes-related PAD, with a propensity to affect tibial arteries, typically poor collateral circulation, high frequency of comorbidities including renal disease, and elevated risks associated with general anaesthesia and of perioperative mortality, supports the patient-specific approach to choice of revascularisation technique recommended by the IWGDF. |
| Geographically remote people | Consistent with recommendation 11, the panel agreed that rapid referral pathways are required to treatment centres offering appropriate assessment (vascular imaging) and where there is expertise in a range of revascularisations procedures where possible. In addition, the panel noted the importance of thorough investigation of both intrinsic factors including patient co-morbidities and extrinsic factors such as access to surgical expertise and appropriate follow-up care to determine the most appropriate procedure. |
| Aboriginal & Torres Strait Islander people | The panel considered that this recommendation was appropriate for Aboriginal and Torres Strait Islander people. The panel agreed that, as with recommendation 11, the importance of established referral pathways developed in conjunction with community-based Aboriginal Health and Medical Services and where the care provision is supported by an Aboriginal Health Worker is integral to optimising patient outcomes. The panel agreed that specific cultural needs including possible reluctance to travel away from country for treatment should be considered in determining the most appropriate treatment for Aboriginal and Torres Strait Islander people. |
| Monitoring & evaluation | Panel monitoring and evaluation advice is as per recommendation 8. |
| Future research priorities | The panel agreed with the IWGDF recommendations for future research. There is a strong need for high quality evidence comparing endovascular and open techniques via randomised controlled trials using pre-defined and standardised outcomes for wound healing and limb salvage (4). Patients with DFU requiring revascularisation have high rates of co-morbidities including cardiovascular and renal disease which affects suitability for revascularisation and increases risk of patient perioperative mortality. The panel agreed with the IWGDF that further research is also required to establish the effectiveness of venous arterialisation for DFU healing and reducing rates of amputation in patients unsuitable for standard revascularisation (24). |

#### Recommendation 14

Ensure that after a revascularisation procedure in a patient with a diabetes-related foot ulcer, the patient is treated by a multidisciplinary team as part of a comprehensive care plan. (Strong; low)

**eTable C14:** Implementation, monitoring and research considerations for Recommendation 14

| Topic | Considerations |
| --- | --- |
| General implementation | The IWGDF Practical guidelines on prevention and management of diabetes-related foot disease reflect the multifaceted nature of DFU development and management and highlight that the restoration of perfusion is only one aspect of this. Other aspects of conservative care including effective offloading and protection of the ulcer, ongoing wound debridement, appropriate management of infection, glycaemic control, and other comorbidities, and patient education, which remain essential components of successful management (55). |
| Geographically remote people | The panel agreed that this recommendation was applicable to geographically remote people In terms of considerations to use this recommendation in geographically remote people the panels’ advice is consistent with recommendations 11 and 12. |
| Aboriginal & Torres Strait Islander people | The panel agreed that this recommendation was applicable to Aboriginal and Torres Strait Islander people but refer the reader to considerations noted for this population in recommendation 11. The panel agreed that specific cultural needs including possible reluctance to travel away from country for treatment should be considered in determining the most appropriate treatment for Aboriginal and Torres Strait Islander people. |
| Monitoring & evaluation | Panel monitoring and evaluation advice is as per recommendation 8. |
| Future research priorities | The panel agreed that future research priorities should include prospective studies in the Australian context across diverse geographical areas and including Aboriginal and Torres Strait Islander populations to evaluate multidisciplinary care access, and DFU healing and limb salvage outcomes. The panel noted that this research aligns with recommendations for monitoring and evaluation. |

#### Recommendation 15

Urgently assess and treat patients with signs or symptoms of PAD and a diabetes-related foot infection, as they are at particularly high risk for major limb amputation. (Strong; moderate**)**

**eTable C15:** Implementation, monitoring and research considerations for Recommendation 15

| Topic | Considerations |
| --- | --- |
| General implementation | There is a limb loss rate of up to 44% at 12 months for diabetes-related patients with foot infection. For untreated infection there is high risk of widespread tissue loss and sepsis -related mortality (15, 56). The panel agreed with the IWGDF that revascularisation should take place promptly following control of significant infection and patient stabilisation and that any further procedures required to restore foot function should be considered after successful revascularisation (24). |
| Geographically remote people | The panel agreed that this recommendation was applicable to geographically remote people In terms of considerations to use this recommendation in geographically remote people the panels’ advice is consistent with recommendations 11 and 12. |
| Aboriginal & Torres Strait Islander people | The panel agreed that this recommendation was applicable to Aboriginal and Torres Strait Islander people but refer the reader to considerations noted for this population in recommendation 11. |
| Monitoring & evaluation | Panel monitoring and evaluation advice is as per recommendation 8 |
| Future research priorities | Consistent with recommendation 14, the panel agreed that prospective evaluation of DFU healing and limb salvage outcomes for patients with PAD, DFU and infection should be undertaken, including in subpopulations where time to access to care may be compromised, for example due to geographic isolation or lack of availability of culturally safe care. |

#### Recommendation 16

Avoid revascularisation in patients in whom, from the patient's perspective, the risk-benefit ratio for the probability of success of the procedure is unfavourable. (Strong; low)

**eTable C16:** Implementation, monitoring and research considerations for Recommendation 16

| Topic | Considerations |
| --- | --- |
| General implementation | There are numerous factors that may render a patient unsuitable for revascularisation. These include poor likelihood of achieving DFU healing or inevitable major amputation, unacceptable risk posed by anaesthesia and the surgical procedure due to the presence of comorbidities, presence of large areas of tissue loss preventing restoration of a functional foot, incapacity for subsequent mobilisation, poor functional status, and short life-expectancy independent of the presenting DFD. The panel agreed with the IWGDF that a decision to choose conservative care over revascularisation should be discussed with the patient in conjunction with a multidisciplinary care team including a vascular surgeon. Evidence of a 50% healing rate for ischaemic DFU in patients unsuitable for revascularisation should also be considered in determining choice of care (46, 47). Further to this, the panel agreed with the IWGDF that where a patient was considered to be unsuitable for revascularisation, other techniques including venous arterialisation or intermittent pneumatic compression therapy offer potential alternative therapies although their effectiveness is largely unsubstantiated. |
| Geographically remote people | The panel agreed that this recommendation was applicable to people in geographically remote locations. Ensuring ease of access to regular ongoing care in the case of conservative treatment should be a priority when developing individual management plans. Use of remote support via telehealth to assist local delivery of care both post revascularisation and in patients that are unsuitable for revascularisation should be considered in areas where there are limited local health services. |
| Aboriginal & Torres Strait Islander people | The panel agreed this recommendation was applicable to Aboriginal and Torres Strait Islander people. Distrust of Western health service delivery models has been documented in Aboriginal and Torres Strait Islander people in remote Australia (57). This is linked to historical and current issues of dispossession and socioeconomic inequality, concern over being removed from family and community for treatment, along with lack of improvement in Aboriginal and Torres Strait Islander health outcomes through a Western model of health care delivery (57). Therefore the panel agreed involvement of Aboriginal and Torres Strait Islander Health Workers and Aboriginal Health and Medical Services and health care providers in discussions relating to vascular intervention, conservative care and subsequent care provision is essential optimising patient outcomes. |
| Monitoring & evaluation | The panel felt there were no specific monitoring implications for this recommendation, however, advise to consider the general monitoring implications for the chapter when implementing this recommendation. |
| Future research priorities | The panel agreed with the IWGDF that investigation of alternate medical therapies to assist with DFU healing in patients unsuitable for revascularisation should be investigated. Specifically novel therapies including biological therapies such as stem cells and blood mononuclear cells should be trialled to determine if these are effective in healing DFU. |

#### Recommendation 17

Provide intensive cardiovascular risk management for any patient with diabetes and an ischaemic foot ulcer, including support for cessation of smoking, treatment of hypertension, control of glycaemia, and treatment with a statin drug as well as low-dose clopidogrel or aspirin. (Strong; low)

**eTable C17:** Implementation, monitoring and research considerations for Recommendation 17

| Topic | Considerations |
| --- | --- |
| General implementation | PAD commonly co-exists with systemic atherosclerosis and underlying generalised endothelial dysfunction due to vascular inflammation and the abnormal metabolic state. Together these changes increase the risk of cardiac-related morbidity and mortality significantly (58). Patients with PAD and DFU have a five-year mortality rate of approximately 50% with the majority of deaths associated with cardiovascular disease (55, 59). This highlights the need for aggressive cardiovascular risk factor modification (e.g. smoking cessation, improved glycaemic and blood pressure and lipid control) in patients with diabetes and PAD and particularly those with DFU. The panel agreed with the IWGDF that all patients with PAD and DFU should be supported to stop smoking, maintain current guideline recommendations for glycaemic and blood pressure control and to take statin and antiplatelet therapy. The panel also agreed with the IWGDF that there is no clear evidence in favour of one antiplatelet agent over another, although agreed that their use individually and in combination is likely to reduce major limb lower limb events and contribute to a reduction in five- year mortality (60, 61).  Combination therapy using antiplatelet and anticoagulants has recently been suggested to be more effective at reducing rates of major limb events than an antiplatelet alone. However this therapy is also associated with increased risk of non-fatal bleeding events and therefore should be used with caution (60). |
| Geographically remote people | Relative geographical isolation may reduce access to available support and health education and promotion services required for successful risk factor modification. Referral to appropriate remote support for patients through telehealth and online services should be a priority for patients in these areas. |
| Aboriginal & Torres Strait Islander people | This recommendation is applicable to Aboriginal and Torres Strait Islander people. Prevalence of risk factors for PAD and cardiovascular disease including smoking and hypertension are significantly higher in the Aboriginal and Torres Strait Islander people than the general population (5, 6). Consistent with this, diabetes-related complications are responsible for almost 70% of preventable hospitalisations for chronic conditions for Aboriginal and Torres Strait Islander people significantly contributing to the seven-fold increased risk of diabetes related mortality in this population (62). Aggressive risk factor modification through lifestyle intervention and pharmacological therapy is therefore essential to reducing cardiovascular events in the population. The panel noted the need for establishment of appropriate care referral pathways and care provision to be co-ordinated through Aboriginal Health and Medical Services and for care provision to be supported by an Aboriginal Health Worker to optimise patient outcomes. |
| Monitoring & evaluation | The panel felt there were no specific monitoring implications for this recommendation, however, advise to consider the general monitoring implications for the chapter when implementing this recommendation. |
| Future research priorities | The panel agreed that future research priorities should include prospective studies in the Australian context across diverse geographical areas and including high risk populations such as Aboriginal and Torres Strait Islander people to evaluate effectiveness of education interventions to reduce cardiovascular risk and assess the related morbidity and mortality outcomes. |

# REFERENCES

1. Williams DT, Harding KG, Price P. An evaluation of the efficacy of methods used in screening for lower-limb arterial disease in diabetes. Diabetes Care. 2005;28(9):2206-10.

2. Dolan NC, Liu K, Criqui MH, Greenland P, Guralnik JM, Chan C, et al. Peripheral artery disease, diabetes, and reduced lower extremity functioning. Diabetes Care. 2002;25(1):113-20.

3. Jude E, Eleftheriadou I, Tentolouris N. Peripheral arterial disease in diabetes—a review. Diabetic Medicine. 2010;27(1):4-14.

4. Forsythe RO, Apelqvist J, Boyko EJ, Fitridge R, Hong JP, Katsanos K, et al. Effectiveness of revascularisation of the ulcerated foot in patients with diabetes and peripheral artery disease: A systematic review. Diabetes/Metabolism Research and Reviews. 2020;36 Suppl 1:e3279.

5. Hoy WE, Kondalsamy-Chennakesavan S, Wang Z, Briganti E, Shaw J, Polkinghorne K, et al. Quantifying the excess risk for proteinuria, hypertension and diabetes in Australian Aborigines: comparison of profiles in three remote communities in the Northern Territory with those in the AusDiab study. Australian and New Zealand Journal of Public Health. 2007;31(2):177-83.

6. Singh TP, Moxon JV, Healy GN, Cadet-James Y, Golledge J. Presentation and outcomes of indigenous Australians with peripheral artery disease. BMC Cardiovascular Disorders. 2018;18(1):94.

7. Maple-Brown L, Cunningham J, Dunne K, Whitbread C, Howard D, Weeramanthri T, et al. Complications of diabetes in urban Indigenous Australians: the DRUID study. Diabetes Research and Clinical Practice. 2008;80(3):455-62.

8. O'Rourke S, Steffen C, Raulli A, Tulip F. Diabetic major amputation in Far North Queensland 1998–2008: What is the Gap for Indigenous patients? Australian Journal of Rural Health. 2013;21(5):268-73.

9. Rayman G, Hassan A, Tooke JE. Blood flow in the skin of the foot related to posture in diabetes mellitus. British Medical Journal (Clinical Research Edition). 1986;292(6513):87-90.

10. Uccioli L, Mancini L, Giordano A, Solini A, Magnani P, Manto A, et al. Lower limb arterio-venous shunts, autonomic neuropathy and diabetic foot. Diabetes Research and Clinical Practice. 1992;16(2):123-30.

11. Vinik AI, Maser RE, Mitchell BD, Freeman R. Diabetic autonomic neuropathy. Diabetes Care. 2003;26(5):1553-79.

12. Vriens B, D'Abate F, Ozdemir B, Fenner C, Maynard W, Budge J, et al. Clinical examination and non‐invasive screening tests in the diagnosis of peripheral artery disease in people with diabetes‐related foot ulceration. Diabetic Medicine. 2018;35(7):895-902.

13. West M, Chuter V, Munteanu S, Hawke F. Defining the gap: a systematic review of the difference in rates of diabetes-related foot complications in Aboriginal and Torres Strait Islander Australians and non-Indigenous Australians. Journal of Foot and Ankle Research. 2017;10(1):48.

14. Morbach S, Furchert H, Gröblinghoff U, Hoffmeier H, Kersten K, Klauke G-T, et al. Long-term prognosis of diabetic foot patients and their limbs: amputation and death over the course of a decade. Diabetes Care. 2012;35(10):2021-7.

15. Prompers L, Huijberts M, Apelqvist J, Jude E, Piaggesi A, Bakker K, et al. High prevalence of ischaemia, infection and serious comorbidity in patients with diabetic foot disease in Europe. Baseline results from the Eurodiale study. Diabetologia. 2007;50(1):18-25.

16. Forsythe RO, Apelqvist J, Boyko EJ, Fitridge R, Hong JP, Katsanos K, et al. Effectiveness of bedside investigations to diagnose peripheral artery disease among people with diabetes mellitus: A systematic review. Diabetes/Metabolism Research and Reviews. 2020;36 Suppl 1:e3277.

17. McAra S, Trevethan R. Measurement of toe-brachial indices in people with subnormal toe pressures: complexities and revelations. Journal of the American Podiatric Medical Association. 2018;108(2):115-25.

18. Casey SL, Lanting SM, Chuter VH. The ankle brachial index in people with and without diabetes: intra-tester reliability. Journal of Foot and Ankle Research. 2020;13:1-6.

19. Aboyans V, Criqui MH, Abraham P, Allison MA, Creager MA, Diehm C, et al. Measurement and interpretation of the ankle-brachial index: a scientific statement from the American Heart Association. Circulation. 2012;126(24):2890-909.

20. Chuter VH, Casey SL. Pre-measurement rest time affects magnitude and reliability of toe pressure measurements. Blood pressure. 2015;24(3):185-8.

21. Pollak EW, Chavis P, Wolfman Jr EF. The effect of postural changes upon the ankle arterial perfusion pressure. Vascular Surgery. 1976;10(4):219-22.

22. Chuter VH, Casey SL. Effect of premeasurement rest time on systolic ankle pressure. Journal of the American Heart Association. 2013;2(4):e000203.

23. Faglia E, Favales F, Quarantiello A, Calia P, Clelia P, Brambilla G, et al. Angiographic evaluation of peripheral arterial occlusive disease and its role as a prognostic determinant for major amputation in diabetic subjects with foot ulcers. Diabetes Care. 1998;21(4):625-30.

24. Hinchliffe RJ, Forsythe RO, Apelqvist J, Boyko EJ, Fitridge R, Hong JP, et al. Guidelines on diagnosis, prognosis, and management of peripheral artery disease in patients with foot ulcers and diabetes (IWGDF 2019 update). Diabetes/Metabolism Research and Reviews. 2020;36 Suppl 1:e3276.

25. Emanuele MA, Buchanan BJ, Abraira C. Elevated leg systolic pressures and arterial calcification in diabetic occlusive vascular disease. Diabetes Care. 1981;4(2):289-92.

26. Aerden D, Massaad D, von Kemp K, van Tussenbroek F, Debing E, Keymeulen B, et al. The ankle-brachial index and the diabetic foot: A troublesome marriage. Annals of Vascular Surgery. 2011;25(6):770-7.

27. Chuter V, Searle A, Barwick A, Golledge J, Leigh L, Oldmeadow C, et al. Estimating the diagnostic accuracy of the ankle–brachial pressure index for detecting peripheral arterial disease in people with diabetes: A systematic review and meta‐analysis. Diabetic Medicine. 2021;38(2):e14379.

28. Lazzarini PA, Ng V, Kinnear EM, Kamp MC, Kuys SS, Hurst C, et al. The Queensland high risk foot form (QHRFF) - is it a reliable and valid clinical research tool for foot disease? Journal of Foot and Ankle Research. 2014;7(1):7-.

29. Lazzarini PA Van Netten JJ, Fitridge R, Kinnear E, Malone M, Perrin BM, Prentice J, Wraight PR. Australian Diabetic Foot Ulcer Minimum Dataset Dictionary. Brisbane: Diabetic Foot Australia, Wound Management Innovation CRC; 2016.

30. National Association of Diabetes Centres (NADC) and Australian Diabetes Society (ADS). NADC Collaborative Interdisciplinary Diabetes High Risk Foot Services Standards Version 1.1 Sydney, Australia: National Association of Diabetes Centres; 2018 [Available from: <https://nadc.net.au/national-standards/>.

31. Barshes NR, Flores E, Belkin M, Kougias P, Armstrong DG, Mills JL, Sr. The accuracy and cost-effectiveness of strategies used to identify peripheral artery disease among patients with diabetic foot ulcers. Journal Vascular Surgery. 2016;64(6):1682-90.e3.

32. Bunte MC, Jacob J, Nudelman B, Shishehbor MH. Validation of the relationship between ankle–brachial and toe–brachial indices and infragenicular arterial patency in critical limb ischemia. Vascular Medicine. 2015;20(1):23-9.

33. Forsythe RO, Apelqvist J, Boyko EJ, Fitridge R, Hong JP, Katsanos K, et al. Performance of prognostic markers in the prediction of wound healing or amputation among patients with foot ulcers in diabetes: A systematic review. Diabetes/Metabolism Research and Reviews. 2020;36:e3278.

34. Ince P, Game FL, Jeffcoate WJ. Rate of healing of neuropathic ulcers of the foot in diabetes and its relationship to ulcer duration and ulcer area. Diabetes Care. 2007;30(3):660-3.

35. Malvezzi L, Castronuovo Jr JJ, Swayne LC, Cone D, Trivino JZ. The correlation between three methods of skin perfusion pressure measurement: radionuclide washout, laser Doppler flow, and photoplethysmography. Journal of Vascular Surgery. 1992;15(5):823-30.

36. Barwick A, Lanting S, Chuter V. Intra-tester and inter-tester reliability of post-occlusive reactive hyperaemia measurement at the hallux. Microvascular Research. 2015;99:67-71.

37. Romanos MT, Raspovic A, Perrin BM. The reliability of toe systolic pressure and the toe brachial index in patients with diabetes. Journal of Foot and Ankle Research. 2010;3(1):1-8.

38. Sonter J, Sadler S, Chuter V. Inter-rater reliability of automated devices for measurement of toe systolic blood pressure and the toe brachial index. Blood Pressure Monitoring. 2015;20(1):47-51.

39. Bhamidipaty V, Dean A, Yap S, Firth J, Barron M, Allard B, et al. Second toe systolic pressure measurements are valid substitutes for first toe systolic pressure measurements in diabetic patients: a prospective study. European Journal of Vascular and Endovascular Surgery. 2015;49(1):77-82.

40. Wang Z, Hasan R, Firwana B, Elraiyah T, Tsapas A, Prokop L, et al. A systematic review and meta-analysis of tests to predict wound healing in diabetic foot. Journal of Vascular Surgery. 2016;63(2):29S-36S. e2.

41. Steffen C, O'Rourke S. Surgical management of diabetic foot complications: the Far North Queensland profile. Australian New Zealand Journal of Surgery. 1998;68(4):258-60.

42. Ewald D, Patel M, Hall G. Hospital separations indicate increasing need for prevention of diabetic foot complications in central Australia. Australian Journal of Rural Health. 2001;9(6):275-9.

43. O'Rourke S, Steffen C, Raulli A, Tulip F. Diabetic major amputation in Far North Queensland 1998-2008: what is the Gap for Indigenous patients? Australian Journal of Rural Health. 2013;21(5):268-73.

44. Lanting SM, Barwick AL, Twigg SM, Johnson NA, Baker MK, Chiu SK, et al. Post-occlusive reactive hyperaemia of skin microvasculature and foot complications in type 2 diabetes. Journal of Diabetes and its Complications. 2017;31(8):1305-10.

45. Schreuder SM, Nieuwdorp M, Koelemay MJW, Bipat S, Reekers JA. Testing the sympathetic nervous system of the foot has a high predictive value for early amputation in patients with diabetes with a neuroischemic ulcer. BMJ Open Diabetes Research and Care. 2018;6(1):e000592.

46. Elgzyri T, Larsson J, Thörne J, Eriksson K-F, Apelqvist J. Outcome of ischemic foot ulcer in diabetic patients who had no invasive vascular intervention. European Journal of Vascular and Endovascular Surgery. 2013;46(1):110-7.

47. Lepäntalo M, Mätzke S. Outcome of unreconstructed chronic critical leg ischaemia. European Journal of Vascular and Endovascular Surgery. 1996;11(2):153-7.

48. Noronen K, Saarinen E, Albäck A, Venermo M. Analysis of the elective treatment process for critical limb ischaemia with tissue loss: diabetic patients require rapid revascularisation. European Journal of Vascular and Endovascular Surgery. 2017;53(2):206-13.

49. Elgzyri T, Larsson J, Nyberg P, Thörne J, Eriksson K-F, Apelqvist J. Early revascularization after admittance to a diabetic foot center affects the healing probability of ischemic foot ulcer in patients with diabetes. European Journal of Vascular and Endovascular Surgery. 2014;48(4):440-6.

50. Sheehan P, Jones P, Caselli A, Giurini JM, Veves A. Percent change in wound area of diabetic foot ulcers over a 4-week period is a robust predictor of complete healing in a 12-week prospective trial. Diabetes Care. 2003;26(6):1879-82.

51. Fiordaliso F, Clerici G, Maggioni S, Caminiti M, Bisighini C, Novelli D, et al. Prospective study on microangiopathy in type 2 diabetic foot ulcer. Diabetologia. 2016;59(7):1542-8.

52. Vinik AI, Erbas T, Park TS, Pierce KK, Stansberry KB. Methods for evaluation of peripheral neurovascular dysfunction. Diabetes Technology & Therapeutics. 2001;3(1):29-50.

53. Barwick AL, Tessier JW, de Jonge XJ, Ivers JR, Chuter VH. Peripheral sensory neuropathy is associated with altered postocclusive reactive hyperemia in the diabetic foot. BMJ Open Diabetes Research and Care. 2016;4(1).

54. Moneta GL, Yeager RA, Lee RW, Porter JM. Noninvasive localization of arterial occlusive disease: a comparison of segmental Doppler pressures and arterial duplex mapping. Journal of Vascular Surgery. 1993;17(3):578-82.

55. Young MJ, McCardle JE, Randall LE, Barclay JI. Improved survival of diabetic foot ulcer patients 1995–2008: possible impact of aggressive cardiovascular risk management. Diabetes Care. 2008;31(11):2143-7.

56. Fisher TK, Scimeca CL, Bharara M, Mills Sr JL, Armstrong DG. A stepwise approach for surgical management of diabetic foot infections. Journal of the American Podiatric Medical Association. 2010;100(5):401-5.

57. Senior K, Chenhall R. Health beliefs and behavior: the practicalities of "looking after yourself" in an Australian aboriginal community. Medical Anthropolology Quarterly. 2013;27(2):155-74.

58. Low Wang CC, Hess CN, Hiatt WR, Goldfine AB. Clinical update: cardiovascular disease in diabetes mellitus: atherosclerotic cardiovascular disease and heart failure in type 2 diabetes mellitus–mechanisms, management, and clinical considerations. Circulation. 2016;133(24):2459-502.

59. Armstrong DG, Swerdlow MA, Armstrong AA, Conte MS, Padula WV, Bus SA. Five year mortality and direct costs of care for people with diabetic foot complications are comparable to cancer. Journal of Foot and Ankle Research. 2020;13(1):16-.

60. Anand SS, Bosch J, Eikelboom JW, Connolly SJ, Diaz R, Widimsky P, et al. Rivaroxaban with or without aspirin in patients with stable peripheral or carotid artery disease: an international, randomised, double-blind, placebo-controlled trial. The Lancet. 2018;391(10117):219-29.

61. Bonaca MP, Bauersachs RM, Anand SS, Debus ES, Nehler MR, Patel MR, et al. Rivaroxaban in peripheral artery disease after revascularization. New England Journal of Medicine. 2020;382(21):1994-2004.

62. Australian Bureau of Statistics. Australian Aboriginal and Torres Strait Islander Health Survey: Biomedical Results, 2012-13 (cat. no. 4727.0.55.003) 2014 [ <https://www.abs.gov.au/ausstats/abs@.nsf/mf/4727.0.55.003>.].
